# 3D-printed biopolymer-based microneedle for enhanced photodynamic therapy in melanoma treatment

**DOI:** 10.3389/fonc.2025.1642448

**Published:** 2025-09-17

**Authors:** Aishat Adejoke Obalola, Heidi Abrahamse, Sathish Sundar Dhilip Kumar

**Affiliations:** Laser Research Centre, University of Johannesburg, Johannesburg, South Africa

**Keywords:** 3D printing, microneedles, melanoma, biopolymers, photodynamic therapy

## Abstract

Melanoma is a highly aggressive cancer with poor prognosis and resistance to many treatments, especially after metastasis. Developing new preventive and adjuvant therapies is critical for improving melanoma outcomes. Photodynamic therapy (PDT) has shown potential in selectively targeting malignant cells while minimizing damage to healthy tissue. However, improving the delivery of photosensitizers (PS) to melanoma cells while reducing systemic toxicity remains a challenge. Microneedles, a transcutaneous drug delivery method, offer advantages such as better patient compliance and easier management compared to traditional methods like intramuscular or intravenous injection. Despite these benefits, manufacturing precise microneedles remains a hurdle. Recent research has focused on 3D printing techniques for creating transdermal drug delivery devices, including microneedles. This review summarizes recent advantages in 3D printed biopolymer-based drug delivery systems using microneedles, evaluates their potential, and discusses the challenges and future prospects of 3D printing in transdermal therapy.

## Graphical Abstract

3D-Printed Biopolymer Microneedles for Enhanced PDT in Melanoma Treatment- Created in BioRender. Dhilip kumar, S. (2025) https://BioRender.com/fear27s

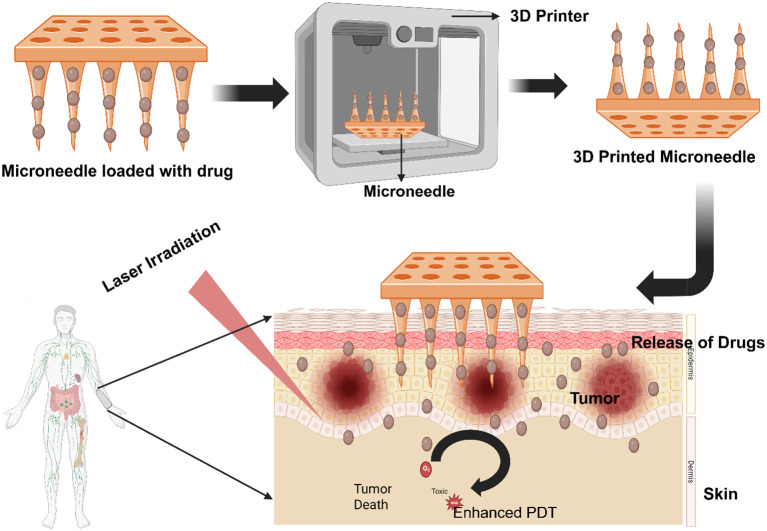

## 1 Introduction

Skin cancer has become a major global health concern, with its incidence steadily increasing, which may significantly affect both the global workforce and economy. The skin has two primary strata: the dermis and the epidermis. The outermost layer of skin, known as the epidermis, comprises Langerhans, Merkel, melanocyte, and keratinocyte cells (1). Cancer is one kind of skin injury that may result from any aberration in this layer. There are two main categories of skin cancer: non-melanoma, which originates from cells originating from the skin’s surface, and melanoma skin cancers, which result from malfunctioning melanocytes (2). Nearly 95% of skin cancer cases are non-melanoma skin cancer (NMSC) (3, 4). Compared to other skin injuries, melanoma represents a negligible fraction of skin malignant tumors—just 1%. Even with recent improvements in treatment methods, melanoma remains the most dangerous kind of skin cancer, with just 15-20% of cases surviving for five years (5).

Oral, intravenous, and intraperitoneal routes are among the traditional techniques for delivering cancer-based medicinal medicines; nevertheless, these routes often have several significant drawbacks (6). Transdermal drug delivery systems (TDDS) offer a non-intrusive, painless method of distribution as opposed to oral and intravenous methods. It is well known to increase the bioavailability of medications with limited permeability and solubility (7, 8). One of the main obstacles to TDDS is the stratum corneum layer, which only allows a few indications to penetrate the skin (9).

To overcome this limitation, scientists have recently developed Microneedle (MN)-based matrices that detect cancer and enable transdermal administration of medications inside the tumor area (10), Although MN-based drug delivery systems were proposed over thirty years ago, their clinical use has recently gained popularity (11). Notwithstanding these unique qualities, the difficulty in accurately producing such micro-scaled devices has significantly impeded their actual industrial applications (12). Remarkably, the advent of three-dimensional Printing (3D printing) technology has effectively addressed the constraints associated with microneedles; this has allowed for the continuous production of one-step products and the development of a desirable microneedle for individualized customization (13, 14).

Additive manufacturing, or 3D printing, is a procedure where a computer-aided design (CAD) module positions materials in layers and selectively creates things with any desired geometric complexity (15, 16). Various 3D printing innovations have been put out in the last ten years to provide adaptable medication doses for a range of uses accurately. These include implants for targeted drug delivery (17, 18) and printlets for oral administration (13). These technologies have great potential in manufacturing pharmaceutical items, especially those with the potential to be individualized treatments (19).

## 2 Principles of photodynamic therapy and its application in melanoma

The most common skin cancer that may be caused by both internal and external sources is melanoma (20). Melanocytes give rise to this kind of cancer. The most common type, cutaneous melanoma, accounts for almost 90–95% of all melanoma occurrences (21) and typically spreads to the brain, eyes, anus, liver, and bone. The degree of involvement and spread to lymph nodes and other nearby healthy tissues determines the stage of melanoma (22). Age, gender, immunodeficiency, family history, and prolonged exposure to ultraviolet radiation (UV) are the most frequent risk factors for melanoma (23). The development of better treatment methods in recent decades has not slowed the sharp increase in melanoma cancer incidence (20). As a result, the concerning increase in MM-related morbidity and death continues to pose a significant obstacle to global healthcare (24). Treatment options for MM include surgery, chemotherapy, radiation, immunotherapy, and molecularly targeted therapy, depending on the patient’s location, stage, and genetics (25). Nevertheless, these therapies often result in unfavorable side effects. While radiation is strongly advised for the treatment of bone, skin, and brain metastases (25), surgery is the primary treatment for early stage melanoma in order to prevent metastasis and improve survival chances (26). Chemotherapeutic medications such dacarbazine (DTIC), temozolomide (TMZ), and fotemustine have been used to treat MM successfully for many years (22). Chemotherapy is still essential for the palliative treatment of tumors that are resistant, progressing, and recurrent, despite the fact that it may have unfavorable effects on nearby normal cells (27). Neutralizing antibodies having a high affinity for immunological blockades, such as programed cell death 1 (PD-1) and cytotoxic T lymphocyte-associated protein 4 (CTLA-4), have improved patient survival rates among immunotherapies (21). Additionally, MM has been significantly impacted by therapies that have target specificity for the oncogenic serine/threonine-protein kinase B-Raf (BRAF) proteins, which are invariably expressed in melanoma patients (21). However, severe patient immunological responses and medication resistance also pose a challenge to these therapy (27). Within eight months of therapy, the majority of patients who have a first and significant tumor recurrence may see a progression in their illness (21). In order to overcome medication resistance and increase the alternatives accessible to MM patients, additional effective medicines are desperately needed (21). In recent years, photodynamic therapy (PDT) has become one of the best cancer treatment options for overcoming the difficulties associated with melanoma (20).

**Figure 1:**
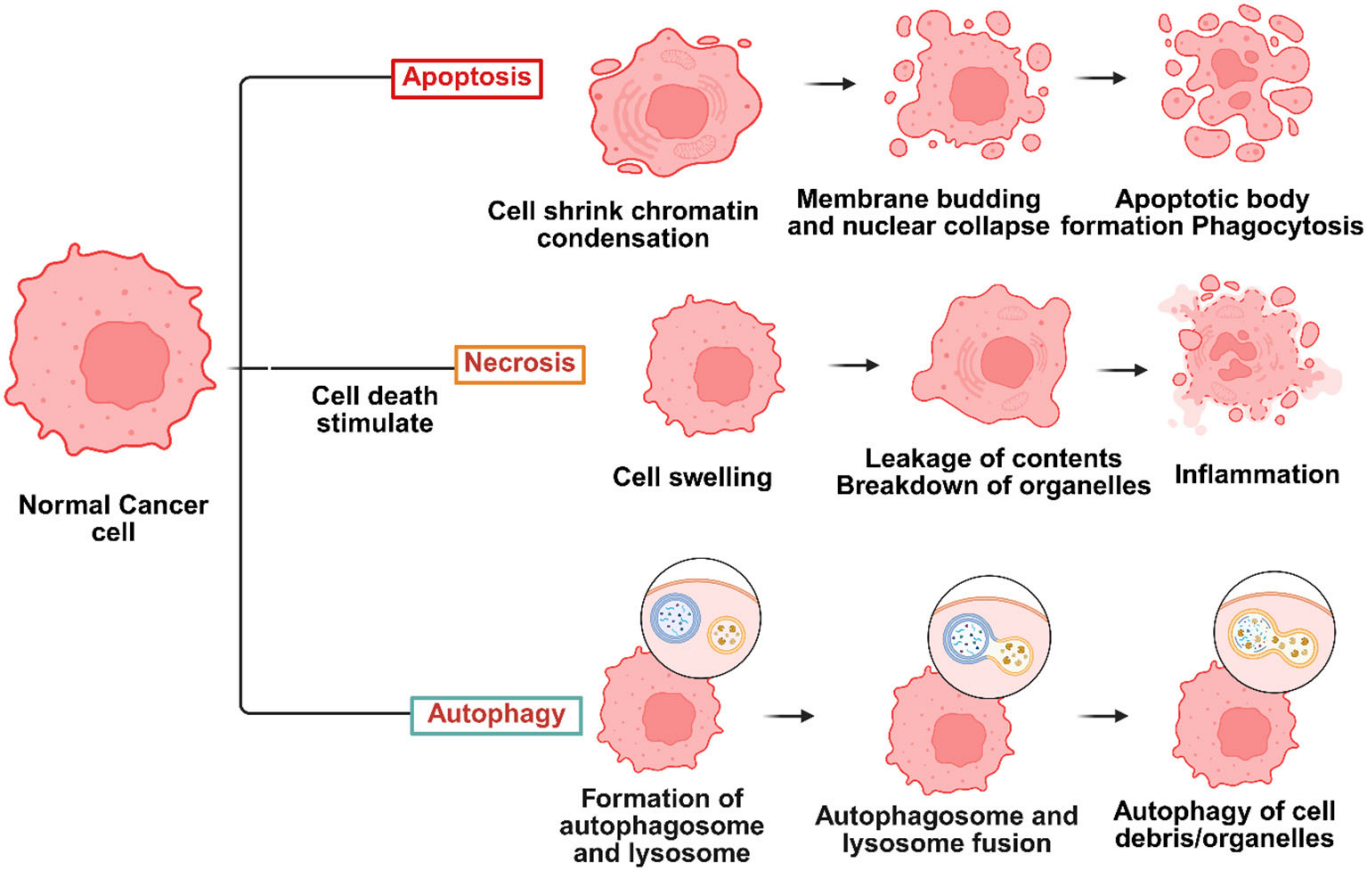
Several types of cell death, including necrosis, autophagy, and apoptosis, may be triggered during photodynamic therapy (PDT) cancer therapies – Created in BioRender. Dhilip kumar, S. (2025) https://BioRender.com/yrtk89u.

Photodynamic therapy (PDT) is a type of phototherapy used to treat cancer (28). It relies on a Photosensitiser (PS), visible light, and the molecular oxygen around the target tissue that coincides with the PS’s absorbance range to cause cellular damage (29). One of thexmain ideas behind PDT is the selective accumulation of photosensitizers in tumor tissues. This happens a lot because of the increased permeability and retention (EPR) effect or receptor-mediated uptake. When photosensitizing agents (PSs) are photoactivated, PDT initiates photochemical processes that destroy a localized tumor in the target area (30). The reactive oxygen species (ROS) produced during the PDT action method cause cell death by necrotic, autophagic, or apoptotic mechanisms (28) (**Figure 1**).

Cell death, known as apoptosis, is regulated and often distinguished by nuclear and membrane deterioration (31). This kind of cell death is the most frequent associated mechanism of cell death in PDT, and the PSs typically localize in cellular mitochondria when it happens (32). Certain signals cause target cells to undergo apoptosis, which sets off several suicide pathways in response to the signals (33). Protein caspases are triggered to break down cellular components, including nucleic and polypeptide material, when the pathways break down (34). As a result, apoptosis is an initiated, controlled process (35). When an external stimulus, such as an infection or trauma, triggers an inflammatory response, necrosis is an unprogramed form of cell death (36). The PS that causes necrosis often localizes inside the target cell’s plasma membrane (37). Membrane permeability and calcium ion transport across the endoplasmic reticulum are components of necrotic cell death pathways (38). Recent research by Dewaele and colleagues (37) has shown that another method of cell death called autophagy may be produced after PDT irradiation of certain PSs. When a cell attempts self-healing to recover from photodamage, it is signaled for programed apoptosis; if this response is unsuccessful, the cell undergoes photodynamic therapy-induced autophagy (39). According to research by Valli and associates, photoactivated zinc phthalocyanine (ZnPcS) showed a higher degree of ROS production, which caused MM cells to undergo necrosis and apoptosis (40). The phototoxicity of Chlorin e6 (Ce6) conjugated to PAMAM dendrimer (generation 7.0) functionalized with RGD peptide was examined in A375 tumor spheroids by Yuan et al. Twelve hours after irradiation, RGD-P-Ce6 produced a noteworthy 25.7% of early apoptotic cells and 25.2% of dead cells (41). A mesoporous nanocarrier containing dabrafenib, phthalocyanine (Pc), was created by Tham et al. In spheroids with 8% cell viability, the nanocomposite demonstrated a much higher cell-killing effectiveness, according to the research (42). Furthermore, PcNP-dug effectively targeted BRAF-positive cancer cells *in vivo* while preserving normal cells that did not express BRAF, achieving 76% tumor shrinkage (42). Some clinical results have been reported, despite ongoing controversy about the therapeutic use of PDT in the treatment of MM. Barbazetto et al. examined four individuals with choroidal melanoma to determine the phototoxicity of verteporfin. According to the findings, melanomas remained resistant and required surgical removal in the remaining instances; however, PDT caused tumor regression in two of them (43). In a similar vein, research conducted by Donaldson et al. examined the effects of verteporfin and laser irradiation in a patient with choroidal amelanotic melanoma and observed a full tumor suppression (44).

### 2.1 Photosensitizers in the treatment of metastatic melanoma

Many PS classes have been studied for PDT therapy of metastatic melanomas **Table 1** (60). There are many things to consider when deciding the kind of PS to use with a certain PDT intervention, including its properties, method of action, localization, and kind of cellular demise it causes. PDT-utilized PSs are divided into three generations according to their photochemical and photophysical characteristics in relation to their cellular mode of action (61). First-generation PSs have significant side effects and often cause vascular tissue damage as their localization, suggesting that their exact location inside the intended cells is constrained (62). Second-generation PS often only has cytotoxic effects on tumor cells (63). To enhance the specific cellular drug absorption and uptake of photosynthetic medicines, third-generation PSs have been supplemented with additional targeted biomolecules (64).

**Table 1:** Investigations of PSs used in cancer PDT.

Photosensitizers	Parameters	Cell model	Outcomes
Phthalocyanine	Wavelength: 630–780 nm; Fluency: 10 J/cm^2^;	Achromic melanoma cells	Notable photo-killing that was linked to lipid peroxidation was observed in cultured cells (45).
Verteporfin	Wavelength: 480 Fluency 0.05–0.18	S91/13 4	Considerable cytotoxicity at a low dosage (46).
Chlorin e6	Wavelength 650 nm Fluency: 10 J/cm^2^	Murine melanoma cell line (B16 cells)	The cell viability with Ce6 and PDT was 22.5% (47).
m-THPC 5	Wavelength 514 nm Fluency: 10–25 J/cm^2^	B16 cells	Overcoming apoptotic inhibitors, PS demonstrated an inhibitory impact in a dose-and energy-intensity-dependent manner (45).
Hypericin	Wavelength 680 Fluency: 1	A375, Mel-1–12 and 501 Mel 13	Necroptosis was caused by severe photodamage to the cell membrane, mitochondria, and endoplasmic reticulum (48).
5-aminolevulinic acid (5-ALA)	Wavelength 628 nm Fluency 20, 40, 60 and 80 J/cm^2^	Mel-Rm	There was a discernible difference between the control group and the groups that received 20, 40, 60, and 80 J/cm^2^ of optical radiation in the presence of 5-ALA (49).
Protoporphyrin IX (PpIX)	Wavelength 630 nm,	Murine melanoma (B16-F10	The findings demonstrate that the energy dosage used directly affects the harmful impact caused by the photoactivation of free PpIX (50).
Protoporphyrin IX (PpIX)	Wavelength 630 nm,	A375 cells	The findings demonstrate that PpIX causes cell death in A375 cells when exposed to light, which is connected to ferroptosis and apoptosis (51).
Verteporfin	Wavelength 650 nm	SK-MEL-28	Verteporfin profoundly alters the behavior of the highly invasive SK-MEL-28, as shown by the marked decline in cell proliferation and the striking alteration in cellular shape (52).
Tetra-4-sulfonatophenyl) porphyrin tetraammonium (TPPS)	Wavelength 405 nm	Mel-Juso	The results of biological experiments indicated that TPPS complexes had a strong anticancer impact, as shown by the decrease of cell adhesion, and suppression of tumor cell growth produced by TPPS when exposed to light (53).
Photoprotoporphyrin IX dimethyl ester (PppIX-DME)	Wavelength 600 nm	B16BL6	When PN-Por was injected into C26 tumor-bearing mice, local light irradiation into the tumor tissues demonstrated a considerable and extremely efficient anti-tumor impact (54).
Porphyrin-zinc(II) triiodide (Zn-EpPor)	Wavelength 430 nm	B16F10 Melanoma	The MTT experiments demonstrated 100% cell death (55).
Y8	Wavelength 808 nm	Human head and neck squamous cell carcinoma	According to the research, the therapy may get rid of primary tumors and strengthen the immune system to fend against metastases (56).
zinc phthalocyanine (ZnPc)	Wavelength 670 nm, 600 mW, 84 mW cm^−2^.	Melanoma cells (B16-F10)	The cytotoxicity finding shows that after light irradiation, B16-F10 cells were more vulnerable to photodamage induced by ZnPc (57).
Chlorin e6	Wavelength 660 nm,	HSC3 cells	Experiments using PTT, PDT, and TPZ sensitised chemotherapy and radiation showed that CPTA had a great therapeutic impact (58).
Aluminum-phthalocyanine	Wavelength 660 m, 25.88 J/cm^2^)	B16-F10	Reactive oxygen species created by the therapy negate the antioxidant effect of melanin produced by the cell, leading to related cell damage (59).

## 3 Transdermal drug delivery system

A collection of physical-chemical innovations that have the potential to control how pharmacologically active substances are released and transported into tissues, cells, and organs such that these active substances can have the greatest possible effects are collectively referred to as drug delivery systems (DDS) (65, 66). Stated differently, DDS include drug delivery methods and formulations that effectively distribute the medicine to enhance therapeutic effectiveness while reducing adverse effects (11).

One of the most researched non-invasive cutaneous drug delivery methods is TDDS. The delivery of many medications has been significantly impacted by TDDS, particularly in the areas of hormone therapy, pain management, and the treatment of conditions affecting the cardiovascular and central nervous systems (67–69). Since TDDS eliminates the need for gastrointestinal tract passage, medications may be administered without pH, enzymes, or intestinal flora interference. It also eliminates loss from first-pass metabolism. Furthermore, TDDS may regulate medication release by consumption limitations, which adds to the method’s high persistence. Most significantly, medications may be safely and conveniently administered to children or the elderly using TDDS, a non-invasive method of delivery that typically causes minimal discomfort to the individual (70, 71).

### 3.1 Patches

When the scopolamine patch was introduced in the 1970s, marking the switch from patchless systems to the TDD patch to achieve a more continuous, regulated, and safe form of drug administration, transdermal drug treatment garnered international interest. The multi-layered drug-storing patch creates a constant medication flow to the skin, enabling continuous administration over an extended length of time. Two different approaches are used to develop patches: the first, known as the “reservoir patch,” has a section where the medication is kept in the proper formulation, and a membrane controls how much is delivered to the skin’s surface. Although Shaw and Theeuwes (1985) found that this design produces release rates that are typically steady, overdose events have been documented because of malfunctioning membranes (72). However, the “matrix patch,” which incorporates the medication evenly into a matrix from which it is delivered to the skin, was created to circumvent the shortcomings of the reservoir-type patch (73). This design may be divided into two primary subcategories: the first uses a polymer that contains a medication, and the second adds an adhesive material to provide a secure fit against the skin. As an alternative, the adhesive—where the medication is contained—is the sole part of the design. At first, TDD patches were quite popular, and many systems based on them were even sold commercially. Commercial patches are available today for a variety of drug administration purposes, including antidepressants, contraceptives, and Alzheimer’s disease treatment (74). However, various impediments prevented the transdermal patch from being widely adopted. According to Hadgraft and Lane (2016), there might be problems with efficacy if a medication crystallizes on the skin before diffusing or if the composition becomes unstable when stored (75).

Consequently, the two patch designs that maximize the percentage of deliveries that can be achieved are the same as the maximum rate that the stratum corneum’s physiology naturally permits. The drawbacks highlight how inappropriate such technologies are for quick, bolus-style medication delivery. Another significant drawback is that these systems depend on the kind of medicine. This restriction has significantly limited the drug palette in the past. It presents substantial obstacles to the transdermal distribution of various medications, including peptides and macromolecules, with greater molecular weights or hydrophilic characteristics (76). Several permeability-enhancing techniques have been developed to overcome these restrictions (77). These techniques use both passive and active ways to increase drug transportation by altering the permeability of the skin. It is noteworthy, however, that the bulk of these methods has been linked to exorbitant expenses, skin irritation, or even discomfort (76, 78). **Table 2** shows different studies on topical patch systems using 3D printing.

**Table 2:** Current studies on transdermal or topical patch systems using 3D printing.

Polymer	3D printing technique	Drug	Studies	Application
Sodium alginate	Syringe Extrusion	Curcumin and chloramphenicol	Biomedical application	Two distinct approaches for regulating drug delivery over time were developed due to *in vitro* drug delivery assays showing that the release functioned quicker in freeze-dried samples than in Ca2+ crosslinked/air-dried ones (79).
Alginate	Semi-solid Extrusion	Propranolol Hydrochloride	Personalized floating formulations	By manufacturing various models, these technologically advanced formulations demonstrated high printing reproducibility and similar release behavior; this innovative process opens the door for their ability to customize medicine doses (80).
Chitosan/Alginate	Freeze deposition	Silver Sulfadiazine	Wound healing	Vertical diffusion Franz cells were used to conduct *in vitro* SSD release studies. As a function of the construct, variations in the amount and release kinetics of medications were observed, which was statistically significant (81).
Alginate/Starch	PAM	Rhodamine B (model drug)	Topical applications	The findings indicated that the quantity of starch added to the solution might affect viscoelastic characteristics (82).
Chitosan methacrylate	Extrusion printing	Lidocaine Hydrochloride and Levofloxacin	Wound healing	These findings demonstrate how different medications can be co-loaded at different sites and drug release rates using 3D Printing (83).
Polyvinyl pyrrolidone (PVP), polyethylene oxide (PEO)	Electrohydrodynamic atomization (EHDA)	Tetracycline hydrochloride	Personalized drug delivery and tailored active release.	Fourier-transform infrared spectroscopy (FTIR) investigations showed that antibiotics were released from patterned structures over five hours compared to current polymer-based matrix systems (84).
Polycaprolactone (PCL)	Coaxial electrohydrodynamic (CEHD)	Tetracycline hydrochloride (TE-HCL)	Drug delivery	According to the research, the CEHD direct-printing approach is a useful on-demand engineering methodology for creating customized patches (85).
Polycaprolactone (PCL)	Electrohydrodynamic	Cellulose acetate-ibuprofen and cellulose acetate-paracetamol	Drug delivery	Two medications with distinct drug release mechanisms were discovered to be delivered via the composite membrane (86).
Pectin	Pneumatic based extrusion	Chitosan and cyclodextrin/propolis extract (CCP) complexes	Wound dressings	Studies conducted *in vitro* demonstrated cytocompatibility, antibacterial activity, and the ability to heal wounds. The addition of the CCP particles improved them (87).
PCL	Hot melt extrusion	Antimicrobial metals	Wound dressings	Research on metal release showed rapid kinetics (up to 24 hours) and a persistent release (up to 72 hours) (88).
Gelatin	Semi-solid extrusion	Doxorubicin	Medical Applications	Patches made with a 3D bioprinter may be used for surgical removal areas or to cure cancerous tissues in patients. They are also beneficial for implantation. (89).
Poly(lactide-*co*-glycolide),	Extrusion	5-fluorouracil	Drug Delivery	The result shows that the local administration of chemotherapeutic medicines to treat tumors might be greatly enhanced by the 3D printing of bioabsorbable implants carrying anti-cancer medications (90).

### 3.2 Microneedles

Microneedles (MN) are arrays of tiny needles arranged in a matrix on its surface. The height of the MNs varies from 25 to 2000 µm, which guarantees their ability to enter the skin’s network of capillaries. Their diameter ensures the device stays away from cutaneous nerve endings and blood arteries. As a result, there is a decreased chance of tissue injury, painless delivery, and infection with microorganisms (19, 91). To maintain the honor of the skin after device removal, the form, size, and material of the MNs must be carefully chosen. Taller MNs have a higher medication loading capacity because of their increased volume. However, patients may experience discomfort from very tall MNs. The ability of the medication to load an array is enhanced by increasing the number of needles in it, but this also results in a proportionate increase in the force needed to insert the MNs. These trade-offs make designing MNs difficult, especially when creating effective, high-quality MNs (92). Based on in-plane and out-of-plane architectures, several varieties of MNs have been created (93). These systems are very patient-friendly because of features like painlessness and self-applying (19).

Furthermore, the shape of MNs allows for more efficient delivery of macromolecules through the skin (94, 95). The lack of resolution, the expensive cost of manufacturing, and localized irritation at the point of application have all been noted as drawbacks of this system despite its popularity as an alternative to standard hypodermic and subcutaneous injections (10). These issues can make it challenging to produce effective MNs on a large scale (93). As previously indicated for patches, changing the mix of the carriers (polymeric substance and medication) may reduce the risk of skin irritation and sensitization.

#### 3.2.1 Types of microneedles

**Figure 2:**
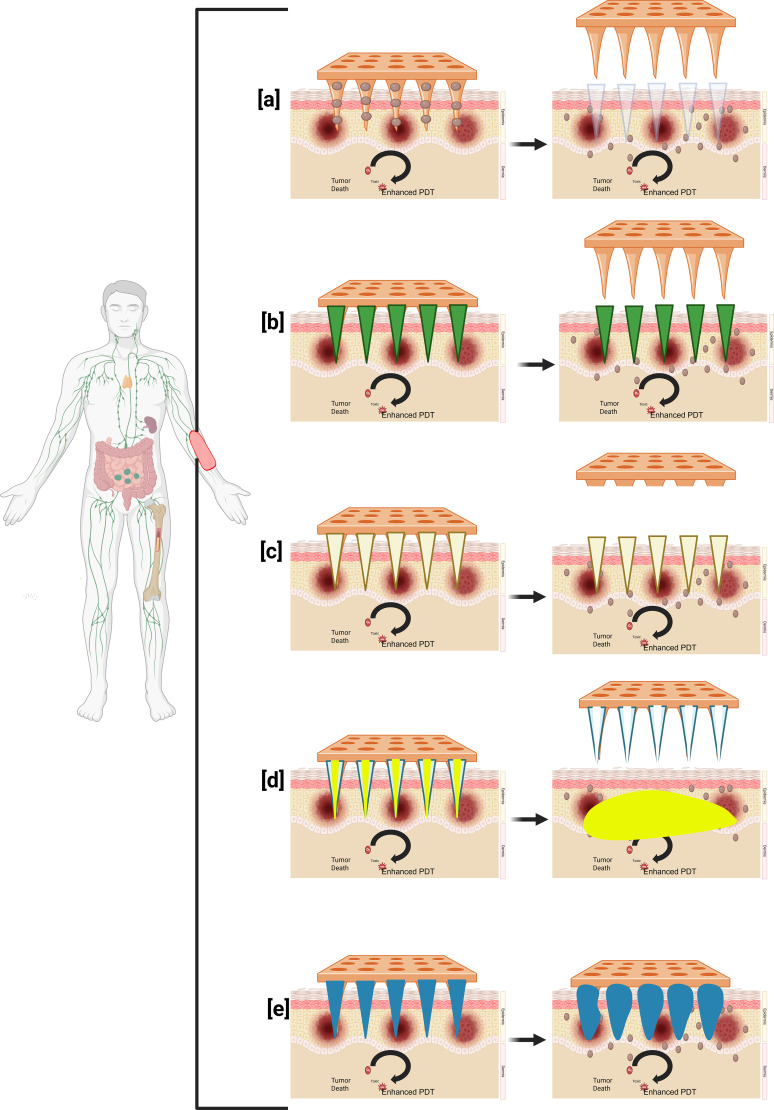
Different types of microneedles. **(a)** Solid removable microneedle, **(b)** Coated microneedle, **(c)** Dissolving microneedle, **(d)** Hollow microneedle, and **(e)** Hydrogel-forming microneedle. Created in BioRender. Dhilip kumar, S. (2025) https://BioRender.com/dg77ew4.

Microneedles are categorized based on their structure, material, and intended use. Each microneedle form has distinct qualities, benefits, drawbacks, uses, and material types. These are a few typical varieties of microneedles **Figure 2**.

##### 3.2.1.1 Solid microneedles

Solid microneedles are mostly used to produce pores on the skin to treat it. The spitzer tips of the needles puncture the skin when a drug patch is placed, creating micron-sized channels that enable the medication to directly penetrate the skin layers and improve penetration; the medication has a systemic impact after being absorbed by the capillaries (96), the medication is applied to epidermal layers via solid microneedles via passive diffusion (97). Biodegradable polymer solid microneedles may improve medication absorption and have enough mechanical strength to penetrate the stratum corneum, according to research on polylactic acid microneedles by Li et al. (96).

##### 3.2.1.2 Coated microneedles

Microneedles with coatings serve two primary purposes. One involves piercing the skin, while the other applies the required medication on the micro needle’s surface. Regrettably, less than 1 milligram is the maximum medication dosage, which explains why coated microneedle development has been restricted (98). Coated microneedles come in a variety of forms that are designed to facilitate drug loading and penetration. Pere et al. created cone and pyramid MN designs for insulin administration using the 3DP approach (99).

##### 3.2.1.3 Hollow microneedles

A hollow chamber that may be utilized for injecting or storing medicine makes up the hollow microneedle (10). Using a non-pressurized drug reservoir as the basis, A route for drug diffusion into the dermis is created using hollow microneedles, an active drug delivery technique. The material composition and manufacturing characteristics of hollow microneedles may be combined to allow for adjustable release kinetics. Depending on the purpose of the application, pharmaceuticals at higher concentrations may provide pharmacological profiles with burst release. In contrast, medications put into a matrix may allow for a medication release that occurs steadily over many days or weeks (100). The hollow microneedle has been used effectively for several vaccinations and vaccines. However, since hollow microneedles are comparatively weaker than solid microneedles and need special attention regarding needle design and insertion technique, they have garnered less attention than solid microneedles. In addition, there are technical issues with the hollow microneedle, such as leaks and blockage while injecting (101).

Long, sharp microneedles that could enter blood arteries for analysis were produced by optimizing the hollow microneedle production process, as described by Li et al. (102). In addition, hollow microneedles exhibit excellent control over drug dosage and release timing despite their challenging manufacturing process that carries hazards of needle breakage and lumen obstruction (10).

##### 3.2.1.4 Biodegradable microneedles

Soluble/biodegradable microneedles offer high loading capacities and complete dissolution upon skin insertion. They also have great biocompatibility. In the first three minutes after applying soluble microneedles to mouse ears, the length of the subcutaneous needles rapidly decreased, After that, the microneedles dissolved steadily but slowly over the next ten minutes. However, this characteristic implies that patients must hold off until the skin’s microneedles disintegrate. It can also be employed for prolonged medication administration (103).

##### 3.2.1.5 Hydrogel microneedles

Hydrogel-based MN drug delivery functions by putting the needle inside the skin, letting the medicine out, and then throwing away the needle. Due to their hydrophilous properties, materials with appropriate swelling characteristics and biocompatibility should be used for hydrogel-based microneedles (97). Aung and colleagues examined the swelling of hydrogel-based microneedles. Even with their little distortion, the microneedles maintained their mechanical strength (104). **Table 3** illustrates current investigations on 3D-printed transdermal microneedle systems.

**Table 3:** Current investigations on 3D-printed transdermal microneedle systems.

Microneedle	Polymer	3D Printing	Drug	Cell	Outcomes
Hollow microneedle	Biocompatible resin material	Liquid crystal display (LCD) vat polymerization	Peptide	Human keratinocytes (HaCaT).	Studies on *in vitro* permeation have shown that HMN seems to be a workable method for peptide administration (105).
	Proprietary class-I resin	Stereolithography	Rifampicin	Male Sprague Dawley rats	Rifampicin delivery using the microneedle reservoir system in SD rats was examined *in vivo*, and the results showed effective penetration and appropriate bioavailability (106).
Solid Microneedles	Biocompatible Class I acrylic resin	Laser Stereolithography	Insulin	Franz diffusion cells	It was shown that coating morphology, fracturing, and piercing behavior were all impacted by the MN shape. It has been shown that MN design affects the way inkjet-coated MNs release insulin to pig skin (91).
	Castable resin	SLA	Calcein and FITCDextran (model dyes)	Human skin	Studies on *in vitro* permeation showed that using solid MNs greatly improved the transport of dyes with and without high molecular weight (107).
Coated Microneedle	Biocompatible Class 1 polymer	SLA	Insulin	Franz diffusion cell	Research *in vitro* and *in vivo* revealed that the coated MN systems released insulin quickly (108).
	PEG	CLIP	Proteins	Female BALB/c mice	Studies conducted *in vivo* on pig skin demonstrated a prompt release of proteins while maintaining catalytic activity. A prolonged protein skin retention MN patch for transdermal protein administration was demonstrated in *in vivo* experiments using mice (109).
	PCL-PVAc-PEG	SLA + IJ	Cisplatin	A-431 cell line	Ex vivo tests on pig skin demonstrated quick drug release as the polymer disintegrated. Mice used in *in vivo* research demonstrated strong anticancer efficacy and tumor remission (110).
Biodegradable microneedle	PLA	FDM	Methylene blue, fluorescein	A431 cells,	Tests conducted *in vitro* revealed that both hues dispersed throughout the skin after MN penetration (111).
Biodegradable microneedle	Sodium alginate	SLA	Vitex agnus-castus	Porcine skin	Both plant extracts had comparable antioxidant and anti-inflammatory qualities *in vivo* tests using obese guinea pigs, but V. agnus-castus had the greatest effects in lowering myeloperoxidase levels (112).

### 3.3 Gel

European Pharmacopoeia defines gels as semisolid preparations for topical application of liquids gelled using an appropriate gelling agent. The gel may be categorized according to a wide range of factors; physical gels, covalently cross-linked gels, and entanglement network gels are the three primary varieties that may be categorized according to the method of cross-linking (113).

Physical hydrogels are dosage forms where the gelling agent is physically cross-linked by hydrogen bonds, electrostatic interaction, or other mechanisms (84). The medium used in physical hydrogels can include water alone or in combination with other polar liquids. These gels are most often characterized by their temperature-dependent sol-gel transition and reversibility (82). Physical gel application may potentially be beneficial for photodynamic treatment (PDT). Photosensitizers are nontoxic substances that may be used topically or orally. When exposed to the right wavelength of light, they can cause visible fluorescence (114). Porphyrins and 5-aminolevulinic acid (5-ALA), its precursor, are well-known examples of photodynamic therapy (PDT) medication. Despite 5-ALA’s tiny size, its hydrophilic nature prevents it from passing through the stratum corneum. The problem may be solved chemically by changing it to a methyl or hexyl ester. Using penetration enhancers like DMSO as an alternative is a possibility (115).

Chemically cross-linked gels make up the second category. Chemical bonds join the cross-linker and the macromolecules in their structure, and these ties are only broken by heat. Because of the higher solvent volume and the flexible polymer chain, these gels have a high elasticity. Three-dimensional swelling networks, covalently cross-linked hydrogels, are created from hydrophilic polymers with different functional groups; these groups can absorb and hold on to enormous volumes of biological fluids and water since they are either grafted or implanted in their structure. Instead of dissolving in water, they inflate and stabilize the medium. The structure may be adjusted by adjusting the hydrogel’s affinity for the aqueous medium and the degree of chemical cross-linking (113, 116).

Polymeric chains interact topologically in a melt or solution to generate or produce polymer entanglement network gels. This is often seen in polymeric gels that comprise one or more higher-molecular-weight polymers, especially elastomers. At frequencies higher than the lifespan of the topological entanglements, they behave like “pseudogels” (117).

### 3.4 Transdermal spray

TS is a topical liquid preparation used as a solution composed of a volatile and non-volatile vehicle containing the fully dissolved medication in solution. Its use allows for better drug penetration through the skin and reaches a sustained level. The potential benefits of TS include improved delivery potential without causing skin irritation due to its nonocclusive nature, increased acceptability, dose flexibility, and ease of manufacture (118). TS is made up of a volatile solvent solution that creates a film that dries quickly when sprayed on the skin (119, 120). The metered dosage transdermal spray (MDTS) system makes sure that the dose is delivered in the right amount from its main packing material. The volatile solvent will deliver the medicine into the top layers of the skin when it is applied, and then it will evaporate. This process leaves a lot of the medication in the skin, which functions as a reservoir to slowly and steadily release the drug into the blood. Once the volatile solvent evaporates from the SC layers, it leaves behind axthin, even coating of the medication that has a lot of thermodynamic activity and quickly gets into the skin (121, 122).

### 3.5 Iontophoresis

A technique known as iontophoresis uses a small electrical current to push a charged drug molecule through the skin (123). The drug molecule’s polarity, valency, mobility, affect iontophoresis effectiveness. Specifically, in contrast to most other drug delivery methods, the reliance on current renders medication absorption via iontophoresis less reliant on biological characteristics (124). This strategy can also include electronic reminders for patients to change their prescriptions as required in order to increase patient compliance (125). Its main advantages are the system’s regulated medication distribution and the ability to be switched on and off as needed. Irritation and discomfort are the system’s limitations, which restrict the drug’s dosage. Now, it is used to provide lidocaine for local anesthesia quickly (126). It’s important to remember that a current of 0.5 mA/cm^2^ or less is safe for the body and might be used (127). When you apply an iontophoretic patch, the drug molecule or the current may sometimes be too strong, which can produce mild to moderate skin redness or irritation (128). But if the current density and exposure duration get up, these little responses might happen more often. There have been complaints in the past of people getting burns or scars on their skin from using the iontophoretic sumatriptan patch. The FDA has looked at these incidents, even if they don’t happen very often. One technique to deal with this issue is to not put a patch over skin that is fractured or injured (127).

### 3.6 Laser radiation

Laser radiation entails exposing the skin to a laser beam to ablate the stratum corneum without harming the epidermis that is still in touch with it. This method of removing the stratum cornea is thought to enhance the administration of lipophilic and hydrophilic medications (129). According to reports, transdermal therapy with laser treatment has benefits, including well-controlled tissue removal, a brief treatment duration, a painless administration route, and minimal side effects. Thus, Norwood Abbey Ltd. has created a handheld portable laser device. In a human volunteer trial, the Norwood Abbey laser device shortened the duration of lidocaine’s start of action to 3–5 minutes, while the control group needed 60 minutes to have a comparable effect (130).

### 3.7 Electroporation

By applying brief, high-voltage electrical pulses to the skin, this novel technique improves medication dispersion by raising permeability. The electrical pulses create microscopic holes in the stratum corneum (SC) used for drug transit. Electrodes placed closely apart introduce electrical pulses to reserve the electric field inside the SC for a painless and safe administration (118). Using hairless mice, it has also been possible to achieve improved transport of bare DNA to the skin *in vivo*; compared to intradermal injection, gene expression was stimulated 100 times more (131).

### 3.8 Ultrasound/sonophoresis

Using a gel, cream, or ointment as a coupling agent, the medicinal component is mixed to transport ultrasonic energy traveling to the skin from the system. To achieve this, the lipids in the stratum cornea must be broken down, allowing the drug to cross the biological barrier (129). Percutaneous absorption is known to be influenced by ultrasound parameters, the most significant of which are frequency, treatment duration, intensity, and intensity (132). In the Katz et al. investigation, dermal anesthesia was achieved after 5 minutes of ultrasonic therapy for skin treated for an average of 9 seconds. This was similar to the 60 minutes needed for skin that had not been treated (133). Smith et al. have also reported on using additional tiny, light new ultrasonic transducers to improve insulin skin transfer *in vitro* (134).

## 4 3D printing

**Figure 3:**
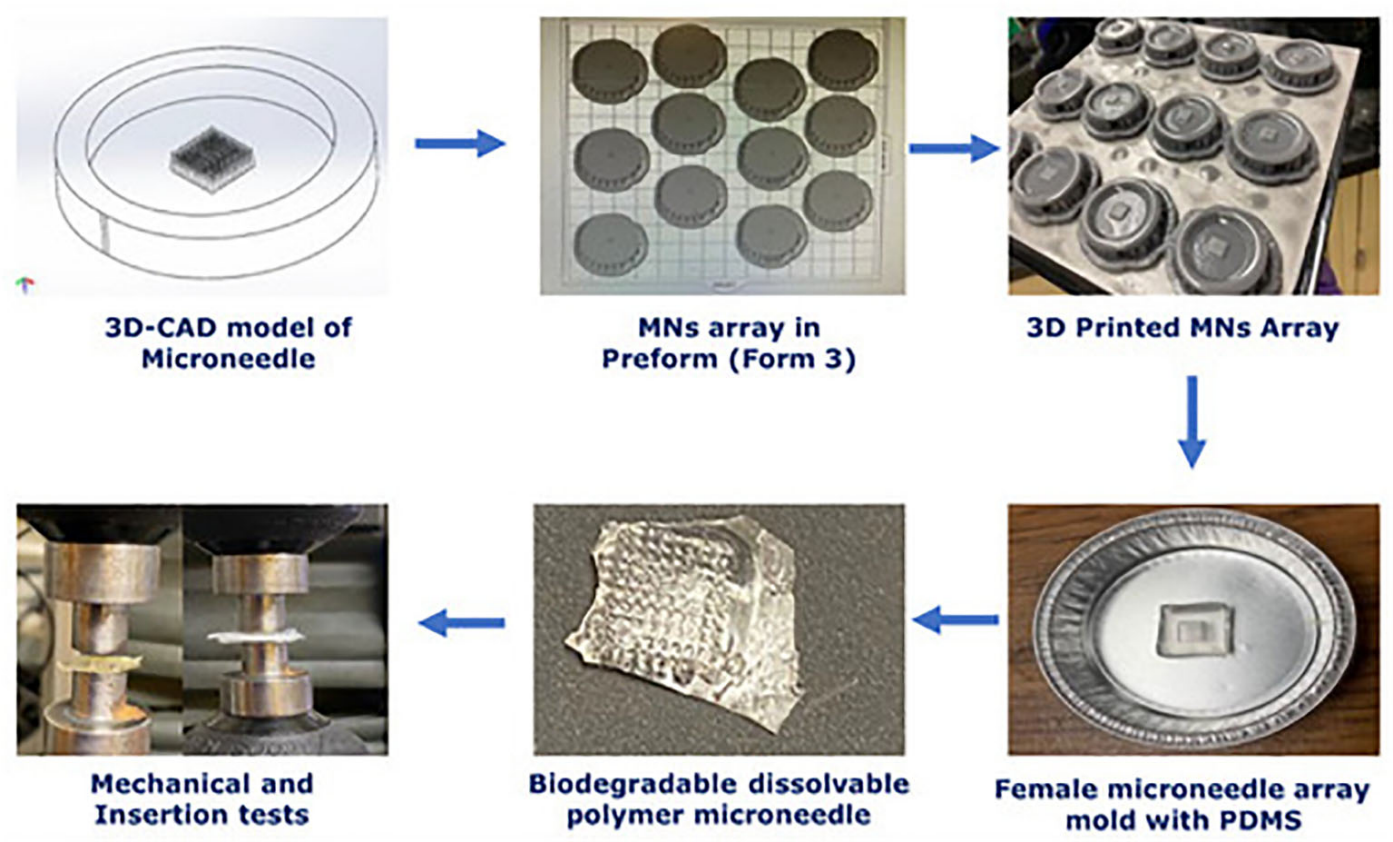
*In vitro* analysis of 3D-printed biopolymer-based microneedle – Adapted from reference (137) under the terms and conditions of the Creative Commons Attribution (CC BY) license.

**Figure 4:**
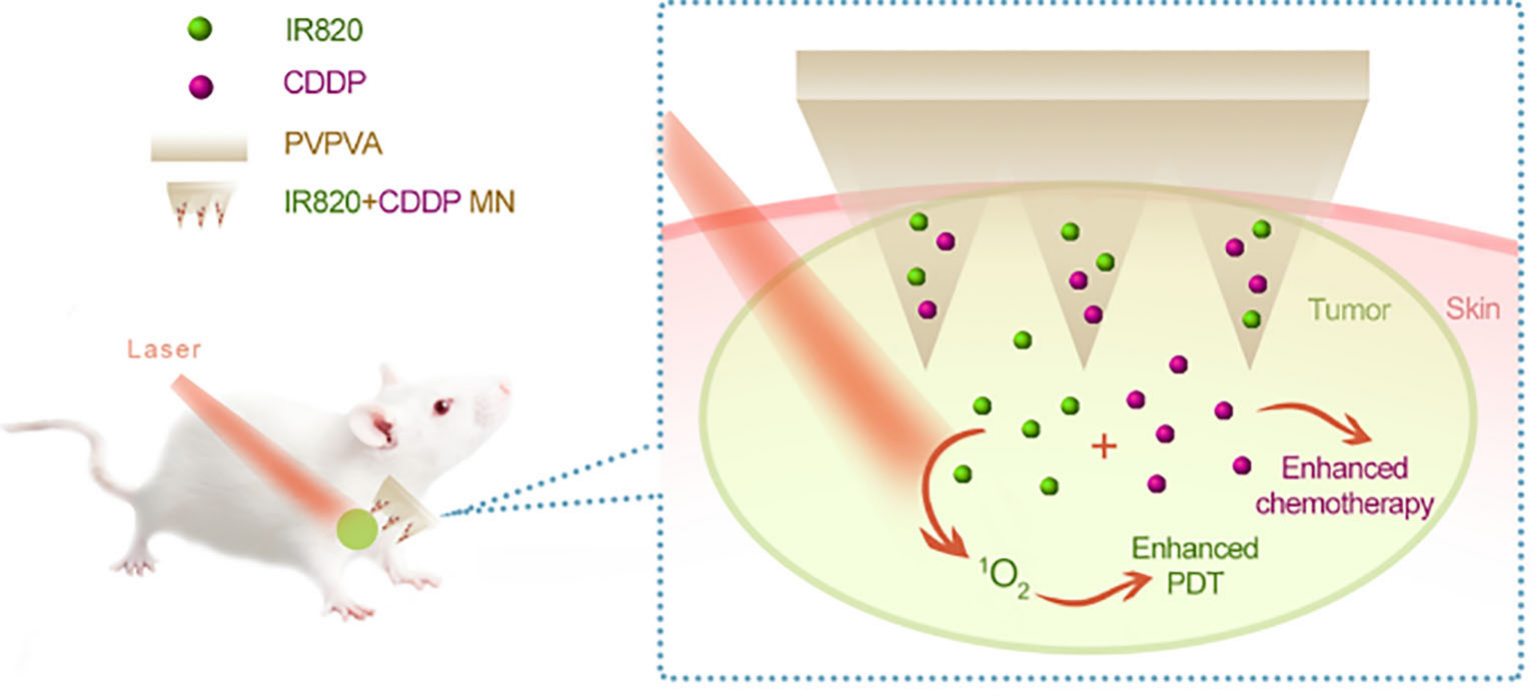
*In vivo* analysis of 3D-printed biopolymer-based microneedle. Adapted from the reference (138) under a Creative Commons Attribution 4.0 International License.

3D printing technology originated with building three-dimensional (3D) structures directly from computer-aided design (CAD) models, layer by layer (135). The technology of 3D Printing is very inventive and has become a flexible technological platform. 3D printing technology is being utilized increasingly in the automotive, aerospace, healthcare, and agricultural sectors to produce open-source designs and mass customization (136). **Figures 3**, **4** show the *in vitro* and *in vivo* application of 3D printing technology.

### 4.1 Categories of 3D printing

The ASTM categorized 3D printing methods into seven classes, Since every technology and machine has a particular set of uses, there are no arguments about which one is superior. These days, 3D printing technologies are increasingly used to produce various goods, not only prototypes (139).

#### 4.1.1 Binder jetting

Powder particles are fused to create a three-dimensional structure via “binder jetting,” where a liquid bonding agent is selectively deposited on the powder particles. The binder jetting technology has many benefits, including huge build volume, fast print speed, cheap cost, free support, and design flexibility. ExOne production printers are a few machines that use the BJ method. The binder jetting method may print on diverse substances, such as polymers, metal, glass, etc (140). A recent optimization study of binder-jetting focused on surface finish and dimensional accuracy. The most significant effects on component shrinkage were caused by layer thickness and binder saturation (141). Another recent work examined the mechanical characteristics of 316 SS lattice structures made by binder jetting and discovered that the elastic modulus was much lower than that of similar AM techniques (142).

#### 4.1.2 Directed energy deposition

Directed energy deposition (DED), a printing technology that is becoming more sophisticated, is often used to improve or fix pre-existing components. High-quality objects may be produced via directed energy deposition, providing excellent control over grain structure. Two examples of this technique are laser deposition and laser-engineered net shaping (LENS) (135). Laser deposition is a scalable technology that may be used to create or repair objects with dimensions ranging from millimeters to meters due to its versatility in a single machine. The oil and gas, aerospace, machining, and transportation sectors are seeing an increase in the usage of this technology. LENS uses thermal energy to melt objects during and after casting (143). Rooyen et al. adopted the DED procedure to fix the fractures in the stainless steel; they discovered that underwater pressure crack sealing of 4.5 and 6.0 mm plate thickness could be effectively fixed with a relatively high power efficiency (144). A marine diesel crankshaft was repaired by Koehler et al. via the DED technique. Based on microstructural analysis, they observed extremely excellent interfacial bonding between the deposited layer and the base metal (145).

#### 4.1.3 Material extrusion

One additive manufacturing method is material extrusion, in which continuous pressure forces the material through a nozzle. When the extruded material exits the nozzle, it will drop onto the substrate steadily and harden completely. To enable the formation of a solid portion and its maintenance in that structure throughout the process, the material also must bond with earlier materials. A few machines based on the ME method include the Fortus Production Series (380, 450, and 900 mc) from Stratasys (146). Periard et al. (7) evaluated many recipes to print sugars via extrusion and printed cake frostings and processed cheeses using Fab@homeTM (147). Co-extrusion was used by Vancauwenberghe et al. (33) to ease worries about the length of time needed for gelling incubation. They created a printer head with an outside layer of CaCl_2_ (a cross-linking solution) and an interior layer of food stimulant based on pectin. Pectin was gelled using this method right during the printing process (148).

#### 4.1.4 Material jetting

For three-dimensional materials, material jetting entails moving the print head and platform in the x, y, and z directions. When the content is sent, for it to print correctly, cross-linking is required. Crosslinking reactions include UV, thermal, ionic, and pH-dependent effects, much as semisolid extrusion. One of this technique’s most significant benefits is the ability to print numerous materials simultaneously, even with distinct characteristics. Bioprinting and small molecules are two applications for material jetting. The droplet size affects the ultimate resolution of material-jet printed prints’ features. Print feature resolution is also strongly influenced by the fluid’s rheological characteristics and print speed, which need precise parameterization. Droplets spread out before they are completely crosslinked as they land on the print, reducing the inkjet’s resolution (149). Coppi et al. used thermal ink jetting to embed human amniotic fluid-derived stem (AFS) cells in an alginate/collagen scaffold. Before being implanted into immunodeficient mice, the printed construct was cultured *in vitro* in an osteogenic medium (150). Michael et al. developed a completely cellularized skin replacement using LIFT. After being put into mice, this construct developed into tissue that resembled basic skin (151). Demirci and Montesano revealed how to use acoustic droplet ejection to encapsulate a single or a small number of cells that were expelled from an open pool. They also demonstrated the potential of this technique to print cells in a variety of biological fluids and hydrogels (152).

#### 4.1.5 Powder bed fusion

In this process, an electron or laser beam is utilized to melt or fuse the material powder. The most common example of this technique is selective laser sintering (SLS). SLS is a quick, high-accuracy 3D printing technique with adjustable surface finishes. SLS uses a powerful laser to sinter powdered polymers to generate a 3D product (153). Irrinki et al.’s study examined the effects of processing settings and powder properties on the corrosion behavior of steel items made using the PBF technology. They discovered that the PBF process components had a higher density (154). With the addition of HA, Tan et al. tested several biocompatible polymers. They proved that such polymers might be used to create TE scaffolds for tissue regeneration (155). Chua et al. created scaffolds for craniofacial and joint abnormalities utilizing SLS and a bio-composite mixture of PVA and HA as a powder material. PVA was chosen because it has strong adhesion, can create complicated forms, and has tensile strength comparable to human articular cartilage. A bioactive substance with osteoconductivity characteristics is HA. HA has the ability to increase the creation of collagen and form links to bones that are as strong as those of a 3- to 6-month-old baby. Examinations of HA’s bioactivity revealed that it remained bioactive in the environment and that the laser sintering procedure had no effect. Therefore, a combination of PVA and HA may create appropriate scaffolds (156).

#### 4.1.6 Sheet lamination

The two primary types of this method are laminated object manufacturing (LOM) and ultrasonic additive manufacturing (UAM), where material sheets are either joined by ultrasound or sliced using a laser. Every sheet of material may be seen as one of the solid object’s cross-sectional layers. The materials used in the SL process are paper, plastic, and metal sheets (157). Wimpenny et al. brazed laser-cut steel sheets to create laminated steel tools with conformal cooling channels (158). In a similar vein, Shuping et al. used a new LOM process with diffusion welding for the rapid manufacturing of metal parts (159).

#### 4.1.7 VAT photopolymerization

The main 3D printing process often used is called photopolymerization, commonly defined as curing photo-reactive polymers using a laser, light, or ultraviolet (UV). Two instances of photopolymerization are digital light processing (DLP) and stereolithography (SLA). Photopolymerization works well for producing luxury goods with exquisite attributes and a smooth surface (160). A biocompatible aliphatic for the DLP process was created by Tzeng et al. After 15 minutes of post-curing, the produced photopolymer had enhanced mechanical characteristics and showed no signs of cytotoxicity (161). Schwartz et al. studied printed polymer constructs resembling anisotropic human hand and multi-material vat photopolymerization. They employed a stiff polymer using dual-wavelength photopolymerization. A soft acrylate network was created by mixing acrylate and epoxy resin and curing it orthogonally with visible light, whereas cationic polymerization (UV light) was used to create the rigid epoxy-acrylate IPN (162).

### 4.2 Materials used for 3D printing

Materials suitable for 3D Printing can be utilized to create desired objects by applying 3D printing technology. Like other production processes, 3D Printing requires improved material properties that adhere to set requirements to generate reliably higher-grade products. This section greatly details the many materials utilized in 3D printing processes (153).

#### 4.2.1 Metals

Due to its strong physical properties, metal may be used in complex manufacturing processes, including printing human organs or aeronautical components. Many industries are showing great interest in metal 3D printing technology because of its advantages (163). These materials include, among others, Cobalt-based alloys that are suitable for use in dental applications that are 3D printed because of their elevated levels of elongation, particular stiffness, resilience, and heat-treated conditions, nickel-base alloys can be used to create aeronautical components as they can tolerate high temperatures and have considerable resistance to corrosion and titanium alloys could also be used by 3D printing technology due to their low density, excellent corrosion, oxidation resistance, and ductility (164, 165).

#### 4.2.2 Polymers

This material is a big molecule of structural units that repeat; natural and manmade polymers fall into this broad category. Due to its superior laser melting and binding properties, nylon is one of the most used and researched polymers. In industry, polymer-based components are often produced “indirectly” by injection molding when produced in medium- and large-scale numbers. Rapid tooling, or AM techniques, may create these molds (166). The work by Nematollahi et al. investigated how the inclusion of polypropylene fiber affected the properties of geopolymers created via 3D printing for use in digital buildings. The researchers’ inclusion of fibers improved pure polypropylene’s ductility, compressive strength, and shape-holding properties (167).

#### 4.2.3 Ceramics

These materials are arduous, brittle, resistant to heat, and erosive. By modifying process settings, 3D printing methods may generate objects with ceramic coatings with no visible holes or cracks, leading to superior mechanical qualities. Because of their fluid condition before curing, ceramics may be utilized to create items with complicated geometry and form; this makes them perfect for buildings and structures, Aeronautical and dental applications can benefit from ceramic materials (168).

3D printing methods may be used to process alumina powder, and this resilient ceramic oxide finds applications in microelectronics, chemicals, adsorbents, and other high-tech fields. Solid bulk ceramics with superior bending characteristics and outstanding compressive strength may be produced via SLCM (Stereolithographic Ceramic Manufacturing) (169)., Li et al. created a porous alumina ceramic that demonstrated an extremely strong flexural elasticity (170). Maurath and Willenbacher showed that a honeycomb structure with a high specific strength and superior dimensional stability may be created by optimizing the ink-printing process (171).

#### 4.2.4 Composites

Composites are materials that comprise many components with various chemical and physical properties. It provides better chemical, physical, and mechanical properties, making them more and more frequent in additive manufacturing due to their accessibility, improved features, and capacity to customize products precisely and economically (172). Currently, composites are used in many Fields, including manufacturing, aerospace, biomedicine, structural, and automotive (173). A bioinspired composite has been published by Leon et al. to enhance the products’ mechanical properties that are 3D printed (174). Digital projection printing was used by Kim et al. to develop an effective piezoelectric nanoparticle-polymer composite material that can be printed into a 3D microstructure (175).

#### 4.2.5 Biomaterials

Biomaterials are the materials most commonly used in the medical field to provide customized solutions. The present research indicates that because of developments in the industry, including enhanced printing accuracy, the ability to print complicated geometry at an inexpensive cost, and reduced material waste, the use of biomaterials in 3D Printing is expanding. Advances in 3D printing technology have enabled noteworthy advancements in health technology, such as implant materials, cell printing, and medical equipment. Tissue engineering, dentistry, and organ model Printing are only a few medical uses for 3D printing technology (176). An integrated tissue-organ printer (ITOP) device that can create human-scale tissue constructions with high mechanical stability in any form was described by Kang et al. (177). Using multi-material 3D bioprinting, Lind et al. presented the creation of a novel class of instrumented cardiac microphysiological devices (178).

### 4.3 Microneedle and PDT applications in melanoma

**Figure 5:**
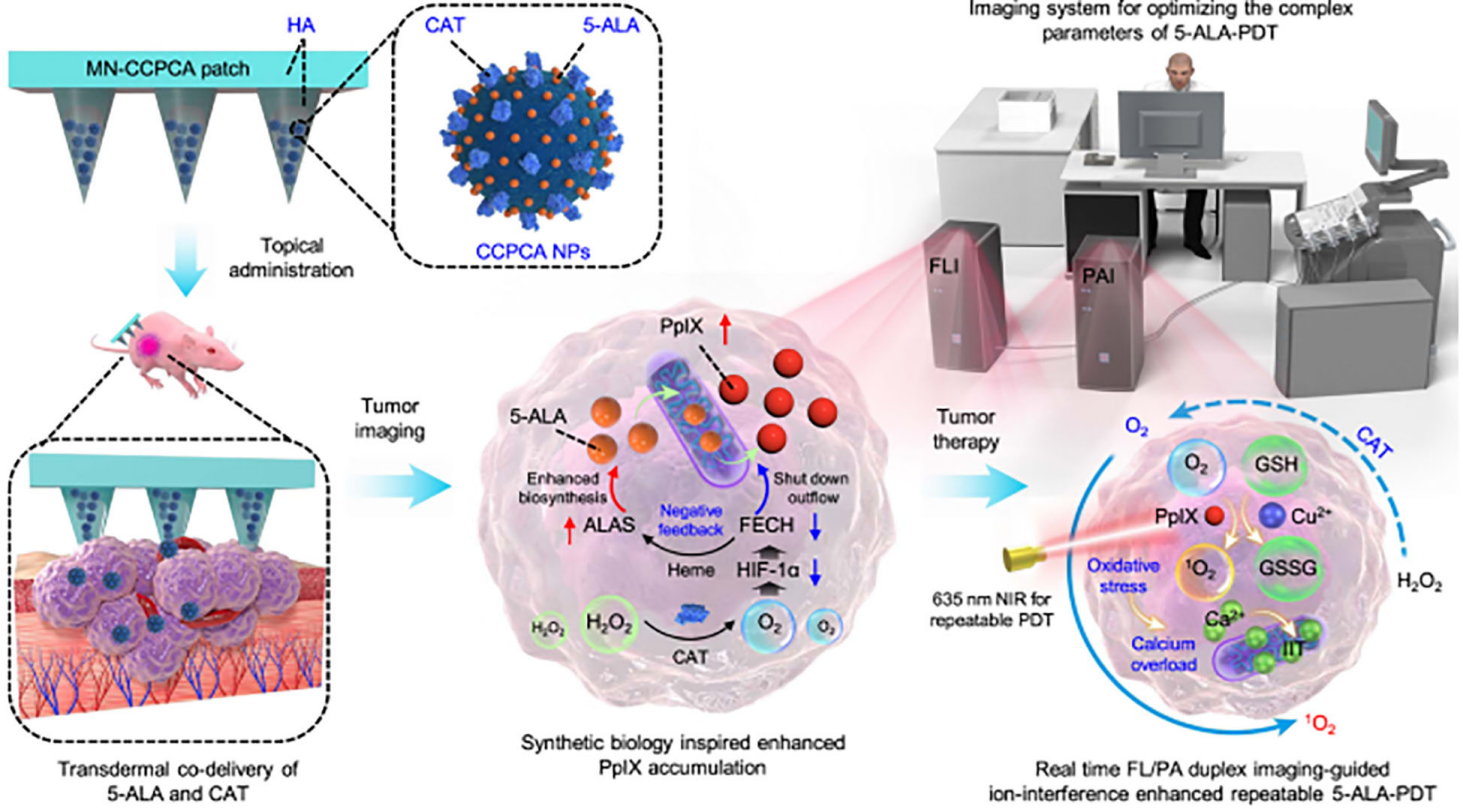
*In vitro* analysis of PDT microneedle – adapted from the reference (180) under a Creative Commons Attribution 4.0 International License.

**Figure 6:**
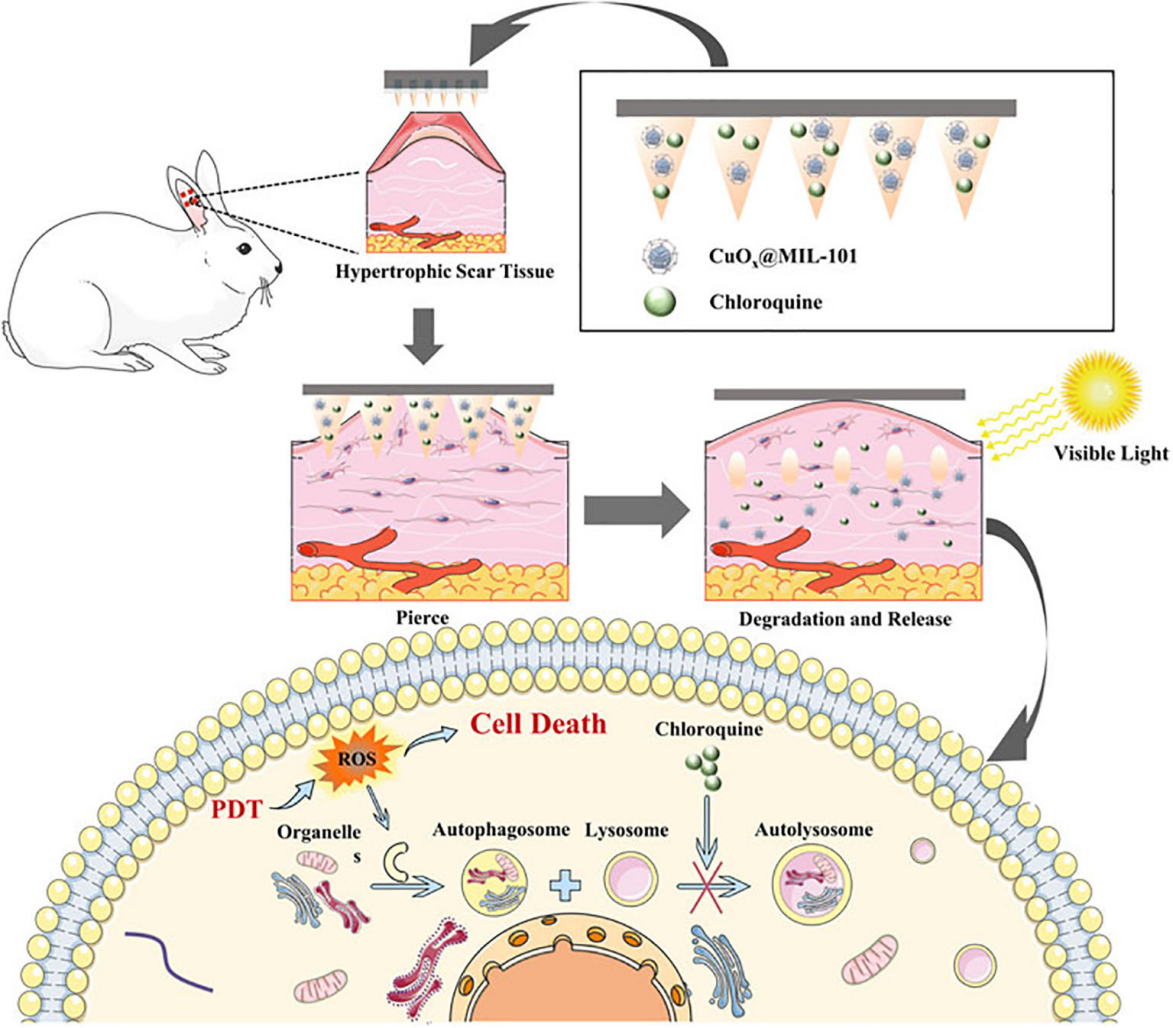
*In vivo* analysis of PDT microneedle – adapted from reference (181) under the terms of the Creative Commons Attribution License (CC BY).

Researchers have been searching for novel treatments to combat melanoma for years. Chemotherapy, immunotherapy, and surgical excision are the mainstays of conventional treatment. However, the traditional methods of administration oral and subcutaneous in these treatments often lead to low levels of drug accumulation. Additionally, systemic exposure to anti-melanoma medications often results in severe side effects that are unpleasant and reduce the effectiveness of treatment. Regarding the management of melanoma, MN is a very novel approach that may increase the transdermal permeability of medications, boost their curative efficacy, and lessen their negative effects. The utilization of MNs as a novel medication delivery mechanism to treat malignant melanoma has drawn interest from several researchers. Melanoma mostly affects the skin. Because the MNs can immediately permeate the skin’s dermis layer, the medication has high permeability, which makes local targeted treatment a reality (179). **Figures 5**, **6** show the *in vitro* and *in vivo* analysis of the PDT Microneedle.

Surgical excision is the mainstay of conventional melanoma therapy; nevertheless, the procedure severely damages the skin. Patients have a poor prognosis, a high risk of recurrence, and a high cost (182). As a result, a novel approach to treating melanoma that is minimally invasive and causes no serious side effects in patients is desperately needed. To accomplish local targeted treatment, MNs provide special benefits when used to treat melanoma as compared to other drug delivery methods.

MNs may directly deliver the medications to the lesion via the skin’s dermis layer. MNs may treat melanoma with minimally invasive procedures and do less harm on the skin. MNs are user-friendly, and their clinical uses will increase patients’ adherence to their prescription regimens (183). Drugs with larger molecular weights and hydrophilicity might be delivered by microneedles to the skin or deeper to the underlying tissues, where they could accumulate locally and have an impact or be released into the systemic circulation (184). The microneedle technology is easy to use, minimally invasive, and economically viable. microneedle arrays are often applied manually or with electric force using a patch, roller, applicator, or injection device in the case of the hollow variety. The process of creating pores or holes in SC either before or in conjunction with applying a medicine to the skin’s surface is the basis for the solid disposable microneedles’ improved drug delivery mechanism. These pores/holes boost the drug’s delivery flow and raise SC’s conductivity. Microneedles come in a variety of shapes and sizes (ranging from 25 to 2000 μm) and are composed of a wide range of materials, including steel, silicon, ceramics, and biodegradable polymers (185). The approach has a wide range of applications due to the described diversity of properties and various drug delivery enhancement processes, which also make microneedles a research focus in the area of transdermal drug administration. Additionally, because of their tiny size, microneedles provide a way to get beyond the SC barrier without hurting or harming the reticular dermal blood vessels (94, 184). Microneedles (MNs) have been explored for treating various skin cancers, including squamous cell carcinoma and basal cell carcinoma (BCC), in addition to malignant melanoma. For instance, Sabri et al. utilized MNs to deliver imiquimod for BCC treatment and compared its efficacy with a commercial topical ointment. Although the MN formulation had lower drug loading, *in vitro* studies showed comparable intradermal penetration to the commercial product (186).

PDT has shown promise for clinical use in melanoma therapy. It may cause cancer cell necrosis by producing a large quantity of reactive oxygen species (ROS) via photosensitizers when exposed to laser light (187). Like chemotherapy medications, photosensitizers are often administered intravenously, which frequently results in systemic damage. Accurate drug release, reduced risk of systemic harm and improved local immune responses are all possible when MNs and PDT are used together (188).

Microneedle technology provides a unique way to administer drugs topically. The stratum corneum may be penetrated by means of hollow microneedles, drug-coated microneedles, drug-encapsulating microneedles, or drug administration on skin perforated with microneedles (189). The application site experiences little discomfort due to the carefully regulated micron-size range of microneedles, and the ability to self-administer for some applications may significantly improve patient compliance (190). Also, to enable precise delivery, the size and form of the microneedle patch may be flexibly modified to meet the uneven shapes of superficial skin melanoma lesions. This is especially important when treating diffuse-type skin melanoma. The possibility of producing systemic toxicity will also be decreased concurrently. (ii) By stimulating the production of proimmune cytokines from epidermal tissues, the MNs may be used as “mechanical adjuvants” to enhance local immune responses (191, 192). Previously, the microneedle-based poke-and-patch method was used to increase dermal penetration of 5-ALA. This method included utilizing microneedles to pierce the skin surface and produce micro-pores before applying a drug-containing patch or formulation (193, 194). A wide range of medications and biomolecules have been delivered to the skin using coated microneedles in recent years, These include medications that are insoluble in water, which are delivered via molten coatings (188, 195).

An oligopeptide hyaluronic acid (oligo-HA) MN patch was created by Bian et al., supplemented with chlorin e6 (Ce6). The MN’s adequate mechanical strength to penetrate the skin barrier facilitated the transport of Ce6 into the deep layer of the skin. The MN made full use of PDT’s effectiveness in preventing the growth of primary and metastatic melanoma under 660 nm laser irradiation. In addition to lowering the expense and danger of systemic toxicity, our Ce6 MN patch improved anti-melanoma immune activity without the need for additional chemotherapeutics or immunological medications, offering a straightforward and useful approach to clinical change (196).

In order to obtain a high killing efficiency of remaining tumor cells after surgery and to increase antitumor immunity in order to avoid tumor recurrence and metastasis, organic PS nanoparticles (N3–4F NPs) were additionally loaded onto the soluble MNs. In *in vivo* breast cancer mice models, it was shown that NPs in the gelatin MNs exhibit superior photothermal conversion efficiency and increased reactive oxygen generation capacity, which results in tumor suppression (197). Li et al. created MNs loaded with catalase and Cu2+ for improved PDT of melanoma by concurrently depleting GSH and producing O2. Fluorescence imaging made it possible to trace the photosensitizers released by the ZIF, allowing for image-guided repeatable PDT for a stronger antitumor impact (198). In order to load dihydroartemisinin (DHA) into the hydrogel network, researchers used acylhydrazone cross-linking to form a connection between the molecule HA-adipic dihydrazide (ADH)-protoporphyrin IX (PpIX) and the “iron reservoir” protocatechualdehyde (PA)-Fe3+ complex in the needle tip. Because of its increased water solubility, HA-ADH-PpIX keeps PpIX from aggregating too much, ensuring effective PDT (199). A study from song et al. showed that both *in vitro* and *in vivo* experiments demonstrate that the CPSA NPs have effective tumor cell toxicity, cholesterol regulation effect, and immunity system evoking ability, which brings a new strategy toward immunosuppression from TME, and paves a new way for adjuvant immunotherapy (200).

For melanoma PDT, ALA-loaded dissolving MNs were also developed, and they showed a stronger anti-tumor effect than ALA injection (201). Silicon MNs-mediated administration of 5-aminolevulinic acid (ALA) was carried out by Donnelly et al.; this method may effectively conduct PDT of skin tumors. ALA released from a bioadhesive patch was considerably enhanced by pretreating the skin with silicon MNs (202). For the photodynamic treatment of melanoma, Tham et al. created mesoporous nanovehicles co-loaded with trametinib, dabrafenib, and phthalocyanine. Additionally, they promoted the transport of nanovehicles into deep tumors for improved antitumor effects using MN technology (42).

## 5 Clinical trial status of microneedle patches for skin cancer treatments

Microneedle-based patches are rapidly advancing in skin cancer research, and the current clinical trial landscape is summarized in **Table 4**.

**Table 4:** Clinical trial status of microneedle-based patches for skin cancer.

ClinicalTrials.Gov Identifier	Name	Application	Phase	Status
NCT05377905	Microneedle Array Doxorubicin (MNA-D)	Cutaneous Squamous Cell Carcinoma	Phase 1and 11	Recruiting
NCT01812837	Microneedle, Aminolevulinic Acid	Actinic Keratosis	NA	Completed
NCT02594644	Microneedle Roller And Aminolevulinic Acid	Keratosis, Actinic	NA	Completed
NCT04928222	Doxorubicin-containing MNA	Basal Cell Carcinoma	Phase 1 and 11	Active, not recruiting
NCT02632110	ALA and Microneedle	Actinic Keratosis	Phase 11	Completed

## 6 Challenges associated with 3D printing

Innovations in 3D Printing provide various advantages throughout medication development and clinical settings. Although it is a powerful technology, its application requires significant adjustments to operations and regulations. Before 3D printing technologies are widely used in clinical settings, several regulatory constraints must be fulfilled, primarily regarding product safety and quality (203).

Material science, life sciences, and clinical science are all closely integrated in the rapidly burgeoning area of 3D printing innovation for health purposes, which will effectively address the organ transplant scarcity problem. Even though cells can already be printed directly, more work must be done before *in vitro* tissue engineering can be accomplished (204). Due to its complexity and many constituent parts, the ECM is challenging to replicate *in vitro* in terms of structure and biological function. Current methods cannot address the problems of oxygen supply and cellular feeding since they mainly stack cell-seeded hydrogels. There are currently insufficient cells available for bigger scaffolds. Compared to cells that adhere to scaffold surfaces, preprophase cells do not get enough nutrition. In other words, the cells exist in 3D space in disequilibrium (204). For printed scaffolds, tissues, and organs to go further, additional constraints about the challenges of cell fusion, differentiation, survival, and development must be met. Material limitations are another issue.

Furthermore, as there are presently no worldwide guidelines for the selection of medicinal materials for 3D printing, assessments based on structure, function, clinical consequences, and other criteria may only be made artificially rather than utilizing trustworthy indicators and enough experimental data. Thus, it will take time and effort to use 3D printing for medical purposes (205, 206).

However, due to 3D Printing, construction automation offers labor-free building. However, further research is needed to make it competitive with more antiquated technology in the mass production of daily items. Studying various preprocessing and postprocessing techniques has shown how crucial surface quality is for 3D-printed parts. Thus, achieving a high-quality surface finish for every size of 3D-printed product is crucial (207).

Adoption in the aviation sector is hampered by factors such as high prices, few resources, and uneven quality of 3D printed parts. Compared to conventional techniques, the main disadvantages of 3D printing in the building sector are its high cost and the possibility of mechanical risk (207).

## 7 Future perspectives and conclusions

Transdermal microneedles have been gaining popularity for various biomedical applications, including medication administration, due to their unique features of little invasion, painlessness, and ease of usage. More remarkably, recent developments in electronic mechanical and intellectual engineering and advancements in photopolymerization have stimulated advances in 3D printing techniques that have made it possible to manufacture artificial and bio-inspired microneedles and patches with complex, functional structures accurately.

Furthermore, the idea of using 3D printing techniques to create microneedles offers exciting possibilities for the administration of certain drugs. More lately, much work has been done to create multifunctional microneedles using various 3D printing methods. The two primary approaches that were the focus of those efforts were photopolymerization and fused deposition modeling. These cutting-edge methods enabled many artificial and naturally inspired printed microneedle designs with ever-more complex structures, much higher printing resolution, and manufacturing accuracy. Another advantage among the technological developments in 3D printing is the growing availability of printing materials and printable pharmaceutical activities. The advancements guarantee that 3D printing technology will soon be able to be essential to the actual manufacture of transdermal microneedles.

Notwithstanding their rapid advancement, the following constraints have a major role in the continued clinical translation of 3D-printed microneedles. Producing precise microneedles requires combining operator experience, material selection, and printing parameter optimization. Even with the steadily increasing availability of printing materials over the last ten years, it makes sense that there are still not enough appropriate formulations, particularly for 3D-printed microneedles, given their mechanical and biological requirements, printing requirements, and possible material toxicity. Furthermore, current technology does not allow for the mass manufacture of 3D-printed goods, particularly for the small-scale manufacture of microneedles and patches. While precision manufacturing and fast prototyping promise to create customized medication with tiny dosages, conventional techniques are still needed for large-scale mass production.

## References

[1] Losquadro WD. Anatomy of the skin and the pathogenesis of nonmelanoma skin cancer. Facial Plast Surg Clinics North America. (2017) 25:283–9. doi: 10.1016/j.fsc.2017.03.001, PMID: 28676156

[2] Esteva A Kuprel B Novoa RA Ko J Swetter SM Blau HM. Dermatologist-level classification of skin cancer with deep neural networks. Nature. (2017) 542:115–8. doi: 10.1038/nature21056, PMID: 28117445 PMC8382232

[3] Didona D Paolino G Bottoni U Cantisani C. Non melanoma skin cancer pathogenesis overview. Biomedicines. (2018) 6:6. doi: 10.3390/biomedicines6010006, PMID: 29301290 PMC5874663

[4] Barton V Armeson K Hampras S Ferris LK Visvanathan K Rollison D. Nonmelanoma skin cancer and risk of all-cause and cancer-related mortality: a systematic review. Arch Dermatol Res. (2017) 309:243–51. doi: 10.1007/s00403-017-1724-5, PMID: 28285366 PMC5396844

[5] Siegel RL Miller KD Jemal A. Cancer statistics, 2018. CA: A Cancer J Clin. (2018) 68:7–30. doi: 10.3322/caac.21442, PMID: 29313949

[6] Dang Y Guan J. Nanoparticle-based drug delivery systems for cancer therapy. In: Smart Materials in Medicine. (2020) 1:10–9. 10.1016/j.smaim.2020.04.001 PMC8455119 34553138

[7] Singh P Carrier A Chen Y Lin S Wang J Cui S. Polymeric microneedles for controlled transdermal drug delivery. J Controlled Release. (2019) 315:97–113. doi: 10.1016/j.jconrel.2019.10.022, PMID: 31644938

[8] Azmana M Mahmood S Hilles AR Mandal UK Saeed Al-Japairai KA Raman S. Transdermal drug delivery system through polymeric microneedle: A recent update. J Drug Delivery Sci Technol. (2020) 60:101877. doi: 10.1016/j.jddst.2020.101877

[9] Yang D Chen M Sun Y Jin Y Lu C Pan X. Microneedle-mediated transdermal drug delivery for treating diverse skin diseases. Acta Biomaterialia. (2021) 121:119–33. doi: 10.1016/j.actbio.2020.12.004, PMID: 33285323

[10] Waghule T Singhvi G Dubey SK Pandey MM Gupta G Singh M. Microneedles: A smart approach and increasing potential for transdermal drug delivery system. Biomedicine Pharmacotherapy. (2019) 109:1249–58. doi: 10.1016/j.biopha.2018.10.078, PMID: 30551375

[11] Li S Gao D Song C Tan C Jiang Y. Isotope labeling strategies for acylcarnitines profile in biological samples by liquid chromatography-mass spectrometry. Analytical Chem. (2019) 91:1701–5. doi: 10.1021/acs.analchem.8b05120, PMID: 30636414

[12] Bilal M Mehmood S Raza A Hayat U Rasheed T Iqbal HMN. Microneedles in smart drug delivery. Adv Wound Care. (2021) 10:204–19. doi: 10.1089/wound.2019.1122, PMID: 32320365 PMC7906867

[13] Ligon SC Liska R Stampfl J Gurr M Mülhaupt R. Polymers for 3D printing and customized additive manufacturing. Chem Rev. (2017) 117:10212–90. doi: 10.1021/acs.chemrev.7b00074, PMID: 28756658 PMC5553103

[14] George E Liacouras P Rybicki FJ Mitsouras D. Measuring and establishing the accuracy and reproducibility of 3D printed medical models. Radiographics. (2017) 37:1424–50. doi: 10.1148/rg.2017160165, PMID: 28800287 PMC5621728

[15] Tay YW Panda B Paul SC Tan MJ Qian SZ Leong KF. Processing and properties of construction materials for 3D printing. Materials Sci Forum. (2016) 861:177–81. doi: 10.4028/www.scientific.net/MSF

[16] Shirazi SFS Gharehkhani S Mehrali M Yarmand H Metselaar HSC Adib Kadri N. A review on powder-based additive manufacturing for tissue engineering: Selective laser sintering and inkjet 3D printing. Science and Technology of Advanced Materials. (2015) 16(3):033502., PMID: 27877783 10.1088/1468-6996/16/3/033502 PMC5099820

[17] Koffler J Zhu W Qu X Platoshyn O Dulin JN Brock J. Biomimetic 3D-printed scaffolds for spinal cord injury repair. Nat Med. (2019) 25:263–9. doi: 10.1038/s41591-018-0296-z, PMID: 30643285 PMC6559945

[18] Cidonio G Glinka M Kim YH Kanczler JM Lanham SA Ahlfeld T. Nanoclay-based 3D printed scaffolds promote vascular ingrowth ex vivo and generate bone mineral tissue *in vitro* and *in vivo* . Biofabrication. (2020) 12:035010. doi: 10.1088/1758-5090/ab8753, PMID: 32259804

[19] Kjar A Huang Y. Application of micro-scale 3D printing in pharmaceutics. Pharmaceutics. (2019) 11:390. doi: 10.3390/pharmaceutics11080390, PMID: 31382565 PMC6723578

[20] Monge-Fuentes V Muehlmann LA de Azevedo RB. Perspectives on the application of nanotechnology in photodynamic therapy for the treatment of melanoma. Nano Rev. (2014) 5:24381–1. doi: 10.3402/nano.v5.24381, PMID: 25317253 PMC4152551

[21] Dhillon SK Porter SL Rizk N Sheng Y McKaig T Burnett K. Rose bengal–amphiphilic peptide conjugate for enhanced photodynamic therapy of Malignant melanoma. J Medicinal Chem. (2020) 63:1328–36. doi: 10.1021/acs.jmedchem.9b01802, PMID: 31940202

[22] Nguyen K Hignett E Khachemoune A. Current and emerging treatment options for metastatic melanoma: a focused review. Dermatol Online J. (2020) 26. doi: 10.5070/D3267049551, PMID: 32898395

[23] Madamsetty VS Paul MK Mukherjee A Mukherjee S. Functionalization of nanomaterials and their application in melanoma cancer theranostics. ACS Biomaterials Sci Eng. (2019) 6:167–81. doi: 10.1021/acsbiomaterials.9b01426, PMID: 33463233

[24] Li X-Y Tan L-C Dong L-W Zhang W-Q Shen X-X Lu X. Susceptibility and resistance mechanisms during photodynamic therapy of melanoma. Front Oncol. (2020) 10:597. doi: 10.3389/fonc.2020.00597, PMID: 32528867 PMC7247862

[25] Domingues B Lopes JM Soares P Pópulo H. Melanoma treatment in review. ImmunoTargets Ther. (2018) 7:35–49. doi: 10.2147/ITT.S134842, PMID: 29922629 PMC5995433

[26] Tambunlertchai S Geary SM Salem AK. Skin penetration enhancement strategies used in the development of melanoma topical treatments. AAPS J. (2021) 23:19. doi: 10.1208/s12248-020-00544-y, PMID: 33404992

[27] Tang JQ Hou XY Yang CS Li YX Xin Y Guo WW. Recent developments in nanomedicine for melanoma treatment. Int J Cancer. (2017) 141:646–53. doi: 10.1002/ijc.30708, PMID: 28340496

[28] Maiuri MC Zalckvar E Kimchi A Kroemer G. Self-eating and self-killing: Crosstalk between autophagy and apoptosis. Nat Rev Mol Cell Biol. (2007) 8:741–52. doi: 10.1038/nrm2239, PMID: 17717517

[29] Nkune NW Abrahamse H. Nanoparticle-based drug delivery systems for photodynamic therapy of metastatic melanoma: A review. Int J Mol Sci. (2021) 22:12549. doi: 10.3390/ijms222212549, PMID: 34830431 PMC8620728

[30] Kumar SSD Abrahamse H. Biocompatible nanocarriers for enhanced cancer photodynamic therapy applications. Pharmaceutics. (2021) 13:1933. doi: 10.3390/pharmaceutics13111933, PMID: 34834348 PMC8624654

[31] Kamal A Faazil S Malik MS. Apoptosis-inducing agents: A patent review (2010-2013). Expert Opin Ther Patents. (2014) 24:339–54. doi: 10.1517/13543776.2014.877445, PMID: 24405450

[32] Mroz P Yaroslavsky A Kharkwal GB Hamblin MR. Cell death pathways in photodynamic therapy of cancer. Cancers. (2011) 3:2516–39. doi: 10.3390/cancers3022516, PMID: 23914299 PMC3729395

[33] Melo-Lima S Gajate C Mollinedo F. Triggers and signaling cross-talk controlling cell death commitment. Cell Cycle. (2015) 14:465–6. doi: 10.1080/15384101.2015.1006540, PMID: 25590143 PMC4347687

[34] Redza-Dutordoir M Averill-Bates DA. Activation of apoptosis signalling pathways by reactive oxygen species. Biochim Biophys Acta - Mol Cell Res. (2016) 1863:2977–92. doi: 10.1016/j.bbamcr.2016.09.012, PMID: 27646922

[35] Holohan C Van Schaeybroeck S Longley DB Johnston PG. Cancer drug resistance: An evolving paradigm. Nat Rev Cancer. (2013), 714–26. doi: 10.1038/nrc3599, PMID: 24060863

[36] Kono H Kimura Y Latz E. Inflammasome activation in response to dead cells and their metabolites. Curr Opin Immunol. (2014) 30:91–8. doi: 10.1016/j.coi.2014.09.001, PMID: 25282339

[37] Dewaele M Martinet W Rubio N Verfaillie T de Witte PA Piette J. Autophagy pathways activated in response to PDT contribute to cell resistance against ROS damage. J Cell Mol Med. (2011) 15:1402–14. doi: 10.1111/j.1582-4934.2010.01118.x, PMID: 20626525 PMC4373339

[38] Belizário J Vieira-Cordeiro L Enns S. Necroptotic cell death signaling and execution pathway: Lessons from knockout mice. Mediators Inflammation. (2015) 2015:128076. doi: 10.1155/2015/128076, PMID: 26491219 PMC4600508

[39] Castano AP Demidova TN Hamblin MR. Mechanisms in photodynamic therapy: Part two - Cellular signaling, cell metabolism and modes of cell death. Photodiagnosis Photodyn Ther. (2005) 2:1–23. doi: 10.1016/S1572-1000(05)00030-X, PMID: 25048553 PMC4108176

[40] Valli F Garcia Vior MC Roguin LP Marino J. Oxidative stress generated by irradiation of a zinc (II) phthalocyanine induces a dual apoptotic and necrotic response in melanoma cells. Apoptosis. (2019) 24:119–34. doi: 10.1007/s10495-018-01512-w, PMID: 30603830

[41] Yuan A Yang B Wu J Hu Y Ming X. Dendritic nanoconjugates of photosensitizer for targeted photodynamic therapy. Acta biomaterialia. (2015) 21:63–73. doi: 10.1016/j.actbio.2015.04.014, PMID: 25900441 PMC4446148

[42] Tham HP Xu K Lim WQ Chen H Zheng M Thng TGS. Microneedle-assisted topical delivery of photodynamically active mesoporous formulation for combination therapy of deep-seated melanoma. ACS nano. (2018) 12:11936–48. doi: 10.1021/acsnano.8b03007, PMID: 30444343

[43] Barbazetto IA Lee TC Rollins IS Chang S Abramson DH. Treatment of choroidal melanoma using photodynamic therapy. Am J Ophthalmol. (2003) 135:898–9. doi: 10.1016/S0002-9394(02)02222-5, PMID: 12788137

[44] Donaldson MJ Lim L Harper CA Mackenzie J Campbell WG. Primary treatment of choroidal amelanotic melanoma with photodynamic therapy. Clin Exp Ophthalmol. (2005) 33:548–9. doi: 10.1111/j.1442-9071.2005.01083.x, PMID: 16181294

[45] Swavey S Tran M. Porphyrin and phthalocyanine photosensitizers as PDT agents: a new modality for the treatment of melanoma. Recent Adv biology Ther Manage melanoma. (2013) 11:253–82. doi: 10.5772/54940

[46] Nowak-Sliwinska P Karocki A Elas M Pawlak A Stochel G Urbanska K. Verteporfin, photofrin II, and merocyanine 540 as PDT photosensitizers against melanoma cells. Biochem Biophys Res Commun. (2006) 349:549–55. doi: 10.1016/j.bbrc.2006.08.060, PMID: 16945338

[47] Yue J Liang L Shen Y Guan X Zhang J Li Z. Investigating dynamic molecular events in melanoma cell nucleus during photodynamic therapy by SERS. Front Chem. (2018) 6:665. doi: 10.3389/fchem.2018.00665, PMID: 30746359 PMC6360157

[48] Kleemann B Loos B Scriba TJ Lang D Davids LM. St John’s Wort (Hypericum perforatum L.) photomedicine: hypericin-photodynamic therapy induces metastatic melanoma cell death. PLoS One. (2014) 9:e103762. doi: 10.1371/journal.pone.0103762, PMID: 25076130 PMC4116257

[49] Mohammadi Z Sazgarnia A Rajabi O Soudmand S Esmaily H Sadeghi HR. An *in vitro* study on the photosensitivity of 5-aminolevulinic acid conjugated gold nanoparticles. Photodiagnosis Photodyn Ther. (2013) 10:382–8. doi: 10.1016/j.pdpdt.2013.03.010, PMID: 24284090

[50] da Silva DB da Silva CL Davanzo NN da Silva Souza R Correa RJ Tedesco AC. (PpIX) loaded PLGA nanoparticles for topical Photodynamic Therapy of melanoma cells. Photodiagnosis Photodyn Ther. (2021) 35:102317. doi: 10.1016/j.pdpdt.2021.102317, PMID: 33940210

[51] Vadarevu H Juneja R Lyles Z Vivero-Escoto JL. Light-activated protoporphyrin IX-based polysilsesquioxane nanoparticles induce ferroptosis in melanoma cells. Nanomaterials. (2021) 11:2324. doi: 10.3390/nano11092324, PMID: 34578640 PMC8470003

[52] Rizzi M Tonello S Estevão BM Gianotti E Marchese L Renò F. Verteporfin based silica nanoparticle for *in vitro* selective inhibition of human highly invasive melanoma cell proliferation. J Photochem Photobiol B: Biol. (2017) 167:1–6. doi: 10.1016/j.jphotobiol.2016.12.021, PMID: 28039784

[53] Nistorescu S Udrea A-M Badea MA Lungu I Boni M Tozar T. Low blue dose photodynamic therapy with porphyrin-iron oxide nanoparticles complexes: *in vitro* study on human melanoma cells. Pharmaceutics. (2021) 13:2130. doi: 10.3390/pharmaceutics13122130, PMID: 34959411 PMC8705854

[54] Ogawara K-i Shiraishi T Araki T Watanabe T-i Ono T Higaki K. Efficient anti-tumor effect of photodynamic treatment with polymeric nanoparticles composed of polyethylene glycol and polylactic acid block copolymer encapsulating hydrophobic porphyrin derivative. Eur J Pharm Sci. (2016) 82:154–60. doi: 10.1016/j.ejps.2015.11.016, PMID: 26593985

[55] Lee KL Carpenter BL Wen AM Ghiladi RA Steinmetz NF. High aspect ratio nanotubes formed by tobacco mosaic virus for delivery of photodynamic agents targeting melanoma. ACS biomaterials Sci Eng. (2016) 2:838–44. doi: 10.1021/acsbiomaterials.6b00061, PMID: 28713855 PMC5509260

[56] Song C Ran J Wei Z Wang Y Chen S Lin L. Organic near-infrared-II nanophotosensitizer for safe cancer phototheranostics and improving immune microenvironment against metastatic tumor. ACS Appl Materials Interfaces. (2021) 13:3547–58. doi: 10.1021/acsami.0c18841, PMID: 33443401

[57] Bolfarini GC Siqueira-Moura MP Demets GJ Morais PC Tedesco AC. *In vitro* evaluation of combined hyperthermia and photodynamic effects using magnetoliposomes loaded with cucurbituril zinc phthalocyanine complex on melanoma. J Photochem Photobiol B: Biol. (2012) 115:1–4. doi: 10.1016/j.jphotobiol.2012.05.009, PMID: 22854225

[58] Song C Xu W Wei Z Ou C Wu J Tong J. Anti-LDLR modified TPZ@ Ce6-PEG complexes for tumor hypoxia-targeting chemo-/radio-/photodynamic/photothermal therapy. J Materials Chem B. (2020) 8:648–54. doi: 10.1039/C9TB02248A, PMID: 31898718

[59] Mello VC Araújo VHS de Paiva KLR Simões MM Marques DC da Silva Costa NR. Development of new natural lipid-based nanoparticles loaded with aluminum-phthalocyanine for photodynamic therapy against melanoma. Nanomaterials. (2022) 12:3547. doi: 10.3390/nano12203547, PMID: 36296737 PMC9609910

[60] Vera RE Lamberti MJ Rivarola VA Rumie Vittar NB. Developing strategies to predict photodynamic therapy outcome: the role of melanoma microenvironment. Tumor Biol. (2015) 36:9127–36. doi: 10.1007/s13277-015-4059-x, PMID: 26419592

[61] Josefsen LB Boyle RW. Photodynamic therapy: Novel third-generation photosensitizers one step closer? Br J Pharmacol. (2008) 154:1–3. doi: 10.1038/bjp.2008.98, PMID: 18362894 PMC2438986

[62] Yoon I Li JZ Shim YK. Advance in photosensitizers and light delivery for photodynamic therapy. Clin Endoscopy. (2013) 46:7–23. doi: 10.5946/ce.2013.46.1.7, PMID: 23423543 PMC3572355

[63] Abrahamse H Hamblin MR. New photosensitizers for photodynamic therapy. Biochem J. (2016) 473:347–64. doi: 10.1042/BJ20150942, PMID: 26862179 PMC4811612

[64] Kataoka H Nishie H Hayashi N Tanaka M Nomoto A Yano S. New photodynamic therapy with next-generation photosensitizers. Ann Trans Med. (2017) 5:. doi: 10.21037/atm.2017.03.59, PMID: 28616398 PMC5464935

[65] Vega-Vasquez P Mosier NS Irudayaraj J. Nanoscale drug delivery systems: from medicine to agriculture. Front Bioeng Biotechnol. (2020) 8:79. doi: 10.3389/fbioe.2020.00079, PMID: 32133353 PMC7041307

[66] Vargason AM Anselmo AC Mitragotri S. The evolution of commercial drug delivery technologies. Nat BioMed Eng. (2021) 5:951–67. doi: 10.1038/s41551-021-00698-w, PMID: 33795852

[67] Roohnikan M Laszlo E Babity S Brambilla D. A snapshot of transdermal and topical drug delivery research in Canada. Pharmaceutics. (2019) 11:256. doi: 10.3390/pharmaceutics11060256, PMID: 31159422 PMC6631132

[68] Pena-Juarez MC Guadarrama-Escobar OR Escobar-Chavez JJ. Transdermal delivery systems for biomolecules. J Pharm Innov. (2022) 17:319–32. doi: 10.1007/s12247-020-09525-2, PMID: 33425065 PMC7786146

[69] Leppert W Malec-Milewska M Zajaczkowska R Wordliczek J. Transdermal and topical drug administration in the treatment of pain. Molecules. (2018) 23:681. doi: 10.3390/molecules23030681, PMID: 29562618 PMC6017304

[70] Akhtar N Singh V Yusuf M Khan RA. Non-invasive drug delivery technology: development and current status of transdermal drug delivery devices, techniques and biomedical applications. BioMed Tech (Berl). (2020) 65:243–72. doi: 10.1515/bmt-2019-0019, PMID: 31926064

[71] Pires LR Vinayakumar KB Turos M Miguel V Gaspar J. A perspective on microneedle-based drug delivery and diagnostics in paediatrics. J Pers Med. (2019) 9:49. doi: 10.3390/jpm9040049, PMID: 31731656 PMC6963643

[72] Mansfield AS Jatoi A. Asphyxiation with a fentanyl patch. Case Rep Oncol. (2013) 6:242–4. doi: 10.1159/000351220, PMID: 23741217 PMC3670640

[73] Margetts L Sawyer R. Transdermal drug delivery: principles and opioid therapy. Continuing Educ Anaesthesia Crit Care Pain. (2007) 7:171–6. doi: 10.1093/bjaceaccp/mkm033

[74] Krestyn E Kolarova H Bajgar R Tomankova K. Photodynamic properties of ZnTPPS(4), ClAlPcS(2) and ALA in human melanoma G361 cells. Toxicol In Vitro. (2010) 24:286–91. doi: 10.1016/j.tiv.2009.08.015, PMID: 19720133

[75] Hadgraft J Lane ME. Drug crystallization - implications for topical and transdermal delivery. Expert Opin Drug Delivery. (2016) 13:817–30. doi: 10.1517/17425247.2016.1140146, PMID: 26766744

[76] Alkilani AZ McCrudden MT Donnelly RF. Transdermal Drug Delivery: Innovative Pharmaceutical Developments Based on Disruption of the Barrier Properties of the stratum corneum. Pharmaceutics. (2015) 7:438–70. doi: 10.3390/pharmaceutics7040438, PMID: 26506371 PMC4695828

[77] Prausnitz MR Langer R. Transdermal drug delivery. Nat Biotechnol. (2008) 26:1261–8. doi: 10.1038/nbt.1504, PMID: 18997767 PMC2700785

[78] Roustit M Blaise S Cracowski JL. Trials and tribulations of skin iontophoresis in therapeutics. Br J Clin Pharmacol. (2014) 77:63–71. doi: 10.1111/bcp.12128, PMID: 23590287 PMC3895348

[79] Olmos-Juste R Guaresti O Calvo-Correas T Gabilondo N Eceiza A. Design of drug-loaded 3D printing biomaterial inks and tailor-made pharmaceutical forms for controlled release. Int J Pharm. (2021) 609:121124. doi: 10.1016/j.ijpharm.2021.121124, PMID: 34597726

[80] Falcone G Saviano M Aquino RP Del Gaudio P Russo P. Coaxial semi-solid extrusion and ionotropic alginate gelation: A successful duo for personalized floating formulations via 3D printing. Carbohydr Polym. (2021) 260:117791. doi: 10.1016/j.carbpol.2021.117791, PMID: 33712139

[81] Bergonzi C Bianchera A Remaggi G Ossiprandi MC Bettini R Elviri L. 3D printed chitosan/alginate hydrogels for the controlled release of silver sulfadiazine in wound healing applications: design, characterization and antimicrobial activity. Micromachines (Basel). (2023) 14:137. doi: 10.3390/mi14010137, PMID: 36677198 PMC9866939

[82] Bom S Santos C Barros R Martins AM Paradiso P Claudio R. Effects of starch incorporation on the physicochemical properties and release kinetics of alginate-based 3D hydrogel patches for topical delivery. Pharmaceutics. (2020) 12:719. doi: 10.3390/pharmaceutics12080719, PMID: 32751818 PMC7466037

[83] Teoh JH Tay SM Fuh J Wang CH. Fabricating scalable, personalized wound dressings with customizable drug loadings via 3D printing. J Control Release. (2022) 341:80–94. doi: 10.1016/j.jconrel.2021.11.017, PMID: 34793918

[84] Wang J-C Chang M-W Ahmad Z Li J-S. Fabrication of patterned polymer-antibiotic composite fibers via electrohydrodynamic (EHD) printing. J Drug Delivery Sci Technol. (2016) 35:114–23. doi: 10.1016/j.jddst.2016.06.009

[85] Yao ZC Wang JC Ahmad Z Li JS Chang MW. Fabrication of patterned three-dimensional micron scaled core-sheath architectures for drug patches. Mater Sci Eng C Mater Biol Appl. (2019) 97:776–83. doi: 10.1016/j.msec.2018.12.110, PMID: 30678967

[86] Wu S Ahmad Z Li JS Chang MW. Fabrication of flexible composite drug films via foldable linkages using electrohydrodynamic printing. Mater Sci Eng C Mater Biol Appl. (2020) 108:110393. doi: 10.1016/j.msec.2019.110393, PMID: 31923982

[87] Andriotis EG Eleftheriadis GK Karavasili C Fatouros DG. Development of bio-active patches based on pectin for the treatment of ulcers and wounds using 3D-bioprinting technology. Pharmaceutics. (2020) 12:56. doi: 10.3390/pharmaceutics12010056, PMID: 31936630 PMC7023266

[88] Muwaffak Z Goyanes A Clark V Basit AW Hilton ST Gaisford S. Patient-specific 3D scanned and 3D printed antimicrobial polycaprolactone wound dressings. Int J Pharm. (2017) 527:161–70. doi: 10.1016/j.ijpharm.2017.04.077, PMID: 28461267

[89] Liu J Tagami T Ozeki T. Fabrication of 3D-printed fish-gelatin-based polymer hydrogel patches for local delivery of pegylated liposomal doxorubicin. Mar Drugs. (2020) 18:325. doi: 10.3390/md18060325, PMID: 32575787 PMC7344981

[90] Yi H-G Choi Y-J Kang KS Hong JM Pati RG Park MN. A 3D-printed local drug delivery patch for pancreatic cancer growth suppression. J Controlled release. (2016) 238:231–41. doi: 10.1016/j.jconrel.2016.06.015, PMID: 27288878

[91] Economidou SN Lamprou DA Douroumis D. 3D printing applications for transdermal drug delivery. Int J Pharmaceutics. (2018) 544:415–24. doi: 10.1016/j.ijpharm.2018.01.031, PMID: 29355656

[92] Johnson AR Caudill CL Tumbleston JR Bloomquist CJ Moga KA Ermoshkin A. Single-step fabrication of computationally designed microneedles by continuous liquid interface production. PloS One. (2016) 11:e0162518. doi: 10.1371/journal.pone.0162518, PMID: 27607247 PMC5015976

[93] Bird D Ravindra NM. Transdermal drug delivery and patches—An overview. Med Devices Sensors. (2020) 3:E10069. doi: 10.1002/mds3.10069

[94] Bariya SH Gohel MC Mehta TA Sharma OP. Microneedles: an emerging transdermal drug delivery system. J Pharm Pharmacol. (2012) 64:11–29. doi: 10.1111/j.2042-7158.2011.01369.x, PMID: 22150668

[95] Park HG Yoon SY Choi JY Lee GS Choi JH Shin CY. Anticonvulsant effect of wogonin isolated from Scutellaria baicalensis. Eur J Pharmacol. (2007) 574:112–9. doi: 10.1016/j.ejphar.2007.07.011, PMID: 17692312

[96] Li J Zeng M Shan H Tong C. Microneedle patches as drug and vaccine delivery platform. Curr Med Chem. (2017) 24:2413–22. doi: 10.2174/0929867324666170526124053, PMID: 28552053

[97] Ita K. Transdermal delivery of drugs with microneedles-potential and challenges. Pharmaceutics. (2015) 7:90–105. doi: 10.3390/pharmaceutics7030090, PMID: 26131647 PMC4588187

[98] Li QY Zhang JN Chen BZ Wang QL Guo XD. A solid polymer microneedle patch pretreatment enhances the permeation of drug molecules into the skin. RSC Adv. (2017) 7:15408–15. doi: 10.1039/C6RA26759A

[99] Pere CPP Economidou SN Lall G Ziraud C Boateng JS Alexander BD. 3D printed microneedles for insulin skin delivery. Int J pharmaceutics. (2018) 544:425–32. doi: 10.1016/j.ijpharm.2018.03.031, PMID: 29555437

[100] . <donnelly2013.pdf>.

[101] Zhang P Dalton C Jullien GA. Design and fabrication of MEMS-based microneedle arrays for medical applications. Microsystem Technol. (2009) 15:1073–82. doi: 10.1007/s00542-009-0883-5

[102] Li CG Lee CY Lee K Jung H. An optimized hollow microneedle for minimally invasive blood extraction. Biomed microdevices. (2013) 15:17–25. doi: 10.1007/s10544-012-9683-2, PMID: 22833155

[103] Zhu DD Zhang XP Zhang BL Hao YY Guo XD. Safety assessment of microneedle technology for transdermal drug delivery: a review. Advanced Ther. (2020) 3:2000033. doi: 10.1002/adtp.202000033

[104] Jina A Tierney MJ Tamada JA McGill S Desai S Chua B. Design, development, and evaluation of a novel microneedle array-based continuous glucose monitor. J Diabetes Sci Technol. (2014) 8:483–7. doi: 10.1177/1932296814526191, PMID: 24876610 PMC4455438

[105] Xenikakis I Tsongas K Tzimtzimis EK Zacharis CK Theodoroula N Kalogianni EP. Fabrication of hollow microneedles using liquid crystal display (LCD) vat polymerization 3D printing technology for transdermal macromolecular delivery. Int J Pharm. (2021) 597:120303. doi: 10.1016/j.ijpharm.2021.120303, PMID: 33540009

[106] Yadav V Sharma PK Murty US Mohan NH Thomas R Dwivedy SK. 3D printed hollow microneedles array using stereolithography for efficient transdermal delivery of rifampicin. Int J Pharm. (2021) 605:120815. doi: 10.1016/j.ijpharm.2021.120815, PMID: 34153441

[107] Xenikakis I Tzimtzimis M Tsongas K Andreadis D Demiri E Tzetzis D. Fabrication and finite element analysis of stereolithographic 3D printed microneedles for transdermal delivery of model dyes across human skin *in vitro* . Eur J Pharm Sci. (2019) 137:104976. doi: 10.1016/j.ejps.2019.104976, PMID: 31254642

[108] Economidou SN Pere CPP Reid A Uddin MJ Windmill JFC Lamprou DA. 3D printed microneedle patches using stereolithography (SLA) for intradermal insulin delivery. Mater Sci Eng C Mater Biol Appl. (2019) 102:743–55. doi: 10.1016/j.msec.2019.04.063, PMID: 31147046

[109] Caudill CL Perry JL Tian S Luft JC DeSimone JM. Spatially controlled coating of continuous liquid interface production microneedles for transdermal protein delivery. J Control Release. (2018) 284:122–32. doi: 10.1016/j.jconrel.2018.05.042, PMID: 29894710

[110] Uddin MJ Scoutaris N Economidou SN Giraud C Chowdhry BZ Donnelly RF. 3D printed microneedles for anticancer therapy of skin tumours. Mater Sci Eng C Mater Biol Appl. (2020) 107:110248. doi: 10.1016/j.msec.2019.110248, PMID: 31761175

[111] Luzuriaga MA Berry DR Reagan JC Smaldone RA Gassensmith JJ. Biodegradable 3D printed polymer microneedles for transdermal drug delivery. Lab Chip. (2018) 18:1223–30. doi: 10.1039/C8LC00098K, PMID: 29536070

[112] Amer RI El-Osaily GH Bakr RO El Dine RS Fayez AM. Characterization and pharmacological evaluation of anti-cellulite herbal product(s) encapsulated in 3D-fabricated polymeric microneedles. Sci Rep. (2020) 10:6316. doi: 10.1038/s41598-020-63271-6, PMID: 32286433 PMC7156484

[113] Nayak AK Das B. Introduction to polymeric gels. Polymeric gels. (2018), 3–27. doi: 10.1016/B978-0-08-102179-8.00001-6

[114] Prausnitz MR. Engineering microneedle patches for vaccination and drug delivery to skin. Annu Rev Chem Biomol Eng. (2017) 8:177–200. doi: 10.1146/annurev-chembioeng-060816-101514, PMID: 28375775

[115] De Rosa FS Bentley MV. Photodynamic therapy of skin cancers: sensitizers, clinical studies and future directives. Pharmaceutical Research. (2000) 17(12):1447–55. doi: 10.1023/A:1007612905378, PMID: 11303952

[116] Capanema NS Mansur AA Carvalho SM Carvalho IC Chagas P de Oliveira LCA. Bioengineered carboxymethyl cellulose-doxorubicin prodrug hydrogels for topical chemotherapy of melanoma skin cancer. Carbohydr polymers. (2018) 195:401–12. doi: 10.1016/j.carbpol.2018.04.105, PMID: 29804993

[117] Kavanagh GM Ross-Murphy SB. Rheological characterisation of polymer gels. Prog Polymer Sci. (1998) 23:533–62. doi: 10.1016/S0079-6700(97)00047-6

[118] Bala P Jathar S Kale S Pal K. Transdermal drug delivery system (TDDS)-a multifaceted approach for drug delivery. J Pharm Res. (2014) 8:1805–35.

[119] Ibrahim SA. Spray-on transdermal drug delivery systems. Expert Opin Drug delivery. (2015) 12:195–205. doi: 10.1517/17425247.2015.961419, PMID: 25227233

[120] Bakshi A Bajaj A Malhotra G Madan M Amrutiya N. A novel metered dose transdermal spray formulation for oxybutynin. Indian J Pharm Sci. (2008) 70:733. doi: 10.4103/0250-474X.49094, PMID: 21369433 PMC3040866

[121] Mandal U. A review on transdermal spray: formulation aspect. MJ Pharma. (2016) 1:006.

[122] Patel D Kumar P Thakkar HP. Lopinavir metered-dose transdermal spray through microporated skin: Permeation enhancement to achieve therapeutic needs. J Drug Delivery Sci Technol. (2015) 29:173–80. doi: 10.1016/j.jddst.2015.07.004

[123] Karamkar PG Agrawal A Chatap VK. A review article: Formulation of topical gel by QbD approach. Adv Pharmacol Pharm. (2023) 11:90–101. doi: 10.13189/app.2023.110202

[124] Jeong WY Kwon M Choi HE Kim KS. Recent advances in transdermal drug delivery systems: a review. Biomater Res. (2021) 25:24. doi: 10.1186/s40824-021-00226-6, PMID: 34321111 PMC8317283

[125] Moarefian M Davalos RV Tafti DK Achenie LE Jones CN. Modeling iontophoretic drug delivery in a microfluidic device. Lab Chip. (2020) 20:3310–21. doi: 10.1039/D0LC00602E, PMID: 32869052 PMC8272289

[126] Kesarwani A Yadav AK Singh S Gautam H Singh HN Sharma A. Theoretical aspects of transdermal drug delivery system. Bull Pharm Res. (2013) 3:78–89.

[127] Bakshi P Vora D Hemmady K Banga AK. Iontophoretic skin delivery systems: Success and failures. Int J Pharm. (2020) 586:119584. doi: 10.1016/j.ijpharm.2020.119584, PMID: 32603836

[128] Curdy C Kalia YN Guy RH. Non-invasive assessment of the effects of iontophoresis on human skin in-vivo. J Pharm Pharmacol. (2001) 53:769–77. doi: 10.1211/0022357011776117, PMID: 11428652

[129] Shingade GM. Review on: recent trend on transdermal drug delivery system. J Drug delivery Ther. (2012) 2:66–75. doi: 10.22270/jddt.v2i1.74

[130] Baron ED Harris L Redpath WS Shapiro H Hetzel F Morley G. Laser-assisted penetration of topical anesthetic in adults. Arch Dermatol. (2003) 139:1288–90. doi: 10.1001/archderm.139.10.1288, PMID: 14568832

[131] Zhang L Nolan E Kreitschitz S Rabussay DP. Enhanced delivery of naked DNA to the skin by non-invasive *in vivo* electroporation. Biochim Biophys Acta (BBA)-General Subj. (2002) 1572:1–9. doi: 10.1016/S0304-4165(02)00270-2, PMID: 12204326

[132] Mitragotri S Kost J. Low-frequency sonophoresis: a review. Advanced Drug delivery Rev. (2004) 56:589–601. doi: 10.1016/j.addr.2003.10.024, PMID: 15019748

[133] Katz N Shapiro D Herrmann T. Ultrasound pretreatment of skin enhances the speed of EMLA cream. Anesth Analg. (2004) 98:371–6. doi: 10.1213/01.ANE.0000099716.02783.C4, PMID: 14742372

[134] Smith NB Lee S Maione E Roy RB McElligott S Shung KK. Ultrasound-mediated transdermal transport of insulin *in vitro* through human skin using novel transducer designs. Ultrasound Med Biol. (2003) 29:311–7. doi: 10.1016/S0301-5629(02)00706-8, PMID: 12659919

[135] Tofail SAM Koumoulos EP Bandyopadhyay A Bose S O’Donoghue L Charitidis C. Additive manufacturing: scientific and technological challenges, market uptake and opportunities. Materials Today. (2018) 21:22–37. doi: 10.1016/j.mattod.2017.07.001

[136] Keleş Ö Blevins CW Bowman KJ. Effect of build orientation on the mechanical reliability of 3D printed ABS. Rapid Prototyping J. (2017) 23:320–8. doi: 10.1108/RPJ-09-2015-0122

[137] Aldawood FK Parupelli SK Andar A Desai S. 3D printing of biodegradable polymeric microneedles for transdermal drug delivery applications. Pharmaceutics. (2024) 16:237. doi: 10.3390/pharmaceutics16020237, PMID: 38399291 PMC10893432

[138] Fu J-j Li C-w Liu Y Chen M-y Zhang Q Yu X-y. The microneedles carrying cisplatin and IR820 to perform synergistic chemo-photodynamic therapy against breast cancer. J nanobiotechnology. (2020) 18:146. doi: 10.1186/s12951-020-00697-0, PMID: 33076924 PMC7574214

[139] Wang Y Blache R Xu X. Selection of additive manufacturing processes. Rapid Prototyping J. (2017) 23:434–47. doi: 10.1108/RPJ-09-2015-0123

[140] Bhushan B Caspers M. An overview of additive manufacturing (3D printing) for microfabrication. Microsystem Technol. (2017) 23:1117–24. doi: 10.1007/s00542-017-3342-8

[141] Chen H Zhao YF. Process parameters optimization for improving surface quality and manufacturing accuracy of binder jetting additive manufacturing process. Rapid Prototyping J. (2016) 22:527–38. doi: 10.1108/RPJ-11-2014-0149

[142] Tang Y Zhou Y Hoff T Garon M Zhao Y. Elastic modulus of 316 stainless steel lattice structure fabricated via binder jetting process. Materials Sci Technol. (2016) 32:648–56. doi: 10.1179/1743284715Y.0000000084

[143] Dilberoglu UM Gharehpapagh B Yaman U Dolen M. The role of additive manufacturing in the era of industry 4.0. Proc Manufacturing. (2017) 11:545–54. doi: 10.1016/j.promfg.2017.07.148

[144] Van Rooyen C Burger H Theron M Doubell P. *In-situ* crack repair by laser cladding. (2010).

[145] Koehler H Partes K Seefeld T Vollertsen F. Laser reconditioning of crankshafts: From lab to application. Phys Proc. (2010) 5:387–97. doi: 10.1016/j.phpro.2010.08.160

[146] Vithani K Goyanes A Jannin V Basit AW Gaisford S Boyd BJ. An overview of 3D printing technologies for soft materials and potential opportunities for lipid-based drug delivery systems. Pharm Res. (2019) 36:1–20. doi: 10.1007/s11095-018-2531-1, PMID: 30406349

[147] Pereira T Barroso S Gil MM. Food texture design by 3D printing: A review. Foods. (2021) 10:320. doi: 10.3390/foods10020320, PMID: 33546337 PMC7913566

[148] Vancauwenberghe V Verboven P Lammertyn J Nicolaï B. Development of a coaxial extrusion deposition for 3D printing of customizable pectin-based food simulant. J Food Eng. (2018) 225:42–52. doi: 10.1016/j.jfoodeng.2018.01.008

[149] Bhattacharjee N Urrios A Kang S Folch A. The upcoming 3D-printing revolution in microfluidics. Lab Chip. (2016) 16:1720–42. doi: 10.1039/C6LC00163G, PMID: 27101171 PMC4862901

[150] De Coppi P Bartsch G Jr. Siddiqui MM Xu T Santos CC Perin L. Isolation of amniotic stem cell lines with potential for therapy. Nat Biotechnol. (2007) 25:100–6. doi: 10.1038/nbt1274, PMID: 17206138

[151] Michael S Sorg H Peck C-T Koch L Deiwick A Chichkov B. Tissue engineered skin substitutes created by laser-assisted bioprinting form skin-like structures in the dorsal skin fold chamber in mice. PloS One. (2013) 8:e57741. doi: 10.1371/journal.pone.0057741, PMID: 23469227 PMC3587634

[152] Demirci U Montesano G. Single cell epitaxy by acoustic picolitre droplets. Lab Chip. (2007) 7:1139–45. doi: 10.1039/b704965j, PMID: 17713612

[153] Shahrubudin N Lee TC Ramlan R. An overview on 3D printing technology: Technological, materials, and applications. Proc manufacturing. (2019) 35:1286–96. doi: 10.1016/j.promfg.2019.06.089

[154] Kibira D Morris KC Kumaraguru S. Methods and tools for performance assurance of smart manufacturing systems. J Res Natl Institute Standards Technol. (2016) 121:282. doi: 10.6028/jres.121.013, PMID: 34434624 PMC7339637

[155] Tan K Chua C Leong K Cheah C Gui W Tan W. Selective laser sintering of biocompatible polymers for applications in tissue engineering. Bio-medical materials Eng. (2005) 15:113–24. doi: 10.1177/0959298920050151-2012 15623935

[156] Chua C Leong K Tan K Wiria F Cheah C. Development of tissue scaffolds using selective laser sintering of polyvinyl alcohol/hydroxyapatite biocomposite for craniofacial and joint defects. J Materials Science: Materials Med. (2004) 15:1113–21. doi: 10.1023/B:JMSM.0000046393.81449.a5, PMID: 15516872

[157] Gibson I Rosen D Stucker B Khorasani M. Additive Manufacturing Technologies. (Vol. 17, pp. 160–86.). Cham, Switzerland: Springer (2021).

[158] Wimpenny DI Bryden B Pashby IR. Rapid laminated tooling. J Materials Process Technol. (2003) 138:214–8. doi: 10.1016/S0924-0136(03)00074-8

[159] Yi S Liu F Zhang J Xiong S. Study of the key technologies of LOM for functional metal parts. Journal of Materials Processing Technology. (2004) 150:175–81.

[160] Low Z-X Chua YT Ray BM Mattia D Metcalfe IS Patterson DA. Perspective on 3D printing of separation membranes and comparison to related unconventional fabrication techniques. J Membrane Sci. (2017) 523:596–613. doi: 10.1016/j.memsci.2016.10.006

[161] Tzeng J-J Yang T-S Lee W-F Chen H Chang H-M. Mechanical properties and biocompatibility of urethane acrylate-based 3D-printed denture base resin. Polymers. (2021) 13:822. doi: 10.3390/polym13050822, PMID: 33800210 PMC7962539

[162] Schwartz J Boydston A. Multimaterial actinic spatial control 3D and 4D printing. Nat Commun. (2019) 10:791. doi: 10.1038/s41467-019-08639-7, PMID: 30770802 PMC6377643

[163] Martin JH Yahata BD Hundley JM Mayer JA Schaedler TA Pollock TM. 3D printing of high-strength aluminium alloys. Nature. (2017) 549:365–9. doi: 10.1038/nature23894, PMID: 28933439

[164] Hitzler L Alifui-Segbaya F Williams P Heine B Heitzmann M Hall W. Additive manufacturing of cobalt-based dental alloys: analysis of microstructure and physicomechanical properties. Adv materials Sci Eng. (2018) 2018:8213023. doi: 10.1155/2018/8213023

[165] Murr LE. Frontiers of 3D printing/additive manufacturing: from human organs to aircraft fabrication. J Materials Sci Technol. (2016) 32:987–95. doi: 10.1016/j.jmst.2016.08.011

[166] Zarringhalam H Majewski C Hopkinson N. Degree of particle melt in Nylon-12 selective laser-sintered parts. Rapid Prototyping J. (2009) 15:126–32. doi: 10.1108/13552540910943423

[167] Nematollahi B Vijay P Sanjayan J Nazari A Xia M Naidu Nerella V. Effect of polypropylene fibre addition on properties of geopolymers made by 3D printing for digital construction. Materials. (2018) 11:2352. doi: 10.3390/ma11122352, PMID: 30469535 PMC6316904

[168] Owen D Hickey J Cusson A Ayeni OI Rhoades J Deng Y. 3D printing of ceramic components using a customized 3D ceramic printer. Prog additive manufacturing. (2018) 3:3–9. doi: 10.1007/s40964-018-0037-3

[169] Tang X Yu Y. Electrospinning preparation and characterization of alumina nanofibers with high aspect ratio. Ceramics Int. (2015) 41:9232–8. doi: 10.1016/j.ceramint.2015.04.157

[170] Li X Gao M Jiang Y. Microstructure and mechanical properties of porous alumina ceramic prepared by a combination of 3–D printing and sintering. Ceramics Int. (2016) 42:12531–5. doi: 10.1016/j.ceramint.2016.05.027

[171] Maurath J Willenbacher N. 3D printing of open-porous cellular ceramics with high specific strength. J Eur Ceramic Soc. (2017) 37:4833–42. doi: 10.1016/j.jeurceramsoc.2017.06.001

[172] Pervaiz S Qureshi TA Kashwani G Kannan S. 3D printing of fiber-reinforced plastic composites using fused deposition modeling: A status review. Materials (Basel). (2021) 14:4520. doi: 10.3390/ma14164520, PMID: 34443044 PMC8399339

[173] Singh S Ramakrishna S Berto F. 3D Printing of polymer composites: A short review. Material Design Process Commun. (2019) 2:E97. doi: 10.1002/mdp2.97

[174] Dimas LS Bratzel GH Eylon I Buehler MJ. Tough composites inspired by mineralized natural materials: computation, 3D printing, and testing. Advanced Funct Materials. (2013) 23:4629–38. doi: 10.1002/adfm.201300215

[175] Kim K Zhu W Qu X Aaronson C McCall WR Chen S. 3D optical printing of piezoelectric nanoparticle–polymer composite materials. ACS nano. (2014) 8:9799–806. doi: 10.1021/nn503268f, PMID: 25046646

[176] Nadagouda MN Rastogi V Ginn M. A review on 3D printing techniques for medical applications. Curr Opin Chem Eng. (2020) 28:152–7. doi: 10.1016/j.coche.2020.05.007 PMC8318092 34327115

[177] Kang H-W Lee SJ Ko IK Kengla C Yoo JJ Atala A. A 3D bioprinting system to produce human-scale tissue constructs with structural integrity. Nat Biotechnol. (2016) 34:312–9. doi: 10.1038/nbt.3413, PMID: 26878319

[178] Lind JU Busbee TA Valentine AD Pasqualini FS Yuan H Yadid M. Instrumented cardiac microphysiological devices via multimaterial three-dimensional printing. Nat materials. (2017) 16:303–8. doi: 10.1038/nmat4782, PMID: 27775708 PMC5321777

[179] Li X Zhao Z Zhang M Ling G Zhang P. Research progress of microneedles in the treatment of melanoma. J Controlled release. (2022) 348:631–47. doi: 10.1016/j.jconrel.2022.06.021, PMID: 35718209

[180] He G Li Y Younis MR Fu L-H He T Lei S. Synthetic biology-instructed transdermal microneedle patch for traceable photodynamic therapy. Nat Commun. (2022) 13:6238. doi: 10.1038/s41467-022-33837-1, PMID: 36266306 PMC9585024

[181] Chen D Zhang Y Long W Chai L Myint TP Zhou W. Visible light-driven photodynamic therapy for hypertrophic scars with MOF armored microneedles patch. Front Chem. (2023) 11:1128255. doi: 10.3389/fchem.2023.1128255, PMID: 36874068 PMC9978826

[182] Sondak VK Gibney GT. Surgical management of melanoma. Hematology/Oncology Clinics. (2014) 28:455–70. doi: 10.1016/j.hoc.2014.02.009, PMID: 24880941

[183] Etzkorn JR Sharkey JM Grunyk JW Shin TM Sobanko JF Miller CJ. Frequency of and risk factors for tumor upstaging after wide local excision of primary cutaneous melanoma. J Am Acad Dermatol. (2017) 77:341–8. doi: 10.1016/j.jaad.2017.03.018, PMID: 28601390

[184] Rzhevskiy AS Singh TRR Donnelly RF Anissimov YG. Microneedles as the technique of drug delivery enhancement in diverse organs and tissues. J Control Release. (2018) 270:184–202. doi: 10.1016/j.jconrel.2017.11.048, PMID: 29203415

[185] Indermun S Luttge R Choonara YE Kumar P du Toit LC Modi G. Current advances in the fabrication of microneedles for transdermal delivery. J Control Release. (2014) 185:130–8. doi: 10.1016/j.jconrel.2014.04.052, PMID: 24806483

[186] Sabri AH Cater Z Gurnani P Ogilvie J Segal J Scurr DJ. Intradermal delivery of imiquimod using polymeric microneedles for basal cell carcinoma. Int J pharmaceutics. (2020) 589:119808. doi: 10.1016/j.ijpharm.2020.119808, PMID: 32891716

[187] Li X Lovell JF Yoon J Chen X. Clinical development and potential of photothermal and photodynamic therapies for cancer. Nat Rev Clin Oncol. (2020) 17:657–74. doi: 10.1038/s41571-020-0410-2, PMID: 32699309

[188] Jain AK Lee CH Gill HS. 5-Aminolevulinic acid coated microneedles for photodynamic therapy of skin tumors. J Controlled Release. (2016) 239:72–81. doi: 10.1016/j.jconrel.2016.08.015, PMID: 27543445

[189] Kim YC Park JH Prausnitz MR. Microneedles for drug and vaccine delivery. Adv Drug Delivery Rev. (2012) 64:1547–68. doi: 10.1016/j.addr.2012.04.005, PMID: 22575858 PMC3419303

[190] Birchall JC Clemo R Anstey A John DN. Microneedles in clinical practice–an exploratory study into the opinions of healthcare professionals and the public. Pharm Res. (2011) 28:95–106. doi: 10.1007/s11095-010-0101-2, PMID: 20238152

[191] Vassilieva EV Wang S Li S Prausnitz MR Compans RW. Skin immunization by microneedle patch overcomes statin-induced suppression of immune responses to influenza vaccine. Sci Rep. (2017) 7:17855. doi: 10.1038/s41598-017-18140-0, PMID: 29259264 PMC5736694

[192] Esser ES Romanyuk A Vassilieva EV Jacob J Prausnitz MR Compans RW. Tetanus vaccination with a dissolving microneedle patch confers protective immune responses in pregnancy. J Control Release. (2016) 236:47–56. doi: 10.1016/j.jconrel.2016.06.026, PMID: 27327766

[193] Mikolajewska P Donnelly RF Garland MJ Morrow DI Singh TR Iani V. Microneedle pre-treatment of human skin improves 5-aminolevulininc acid (ALA)- and 5-aminolevulinic acid methyl ester (MAL)-induced PpIX production for topical photodynamic therapy without increase in pain or erythema. Pharm Res. (2010) 27:2213–20. doi: 10.1007/s11095-010-0227-2, PMID: 20676735

[194] Donnelly RF Raj Singh TR Woolfson AD. Microneedle-based drug delivery systems: microfabrication, drug delivery, and safety. Drug Delivery. (2010) 17:187–207. doi: 10.3109/10717541003667798, PMID: 20297904 PMC2906704

[195] Ma Y Gill HS. Coating solid dispersions on microneedles via a molten dip-coating method: development and *in vitro* evaluation for transdermal delivery of a water-insoluble drug. J Pharm Sci. (2014) 103:3621–30. doi: 10.1002/jps.24159, PMID: 25213295 PMC4374630

[196] Bian Q Huang L Xu Y Wang R Gu Y Yuan A. A facile low-dose photosensitizer-incorporated dissolving microneedles-based composite system for eliciting antitumor immunity and the abscopal effect. ACS Nano. (2021) 15:19468–79. doi: 10.1021/acsnano.1c06225, PMID: 34859990

[197] Song C Wu X Wang J Liu R Zhao Y. Photosensitizer-immunotherapy integrated microneedles for preventing tumor recurrence and metastasis. Nano Today. (2023) 51:101913. doi: 10.1016/j.nantod.2023.101913

[198] Li Y He G Fu L-H Younis MR He T Chen Y. A microneedle patch with self-oxygenation and glutathione depletion for repeatable photodynamic therapy. ACS nano. (2022) 16:17298–312. doi: 10.1021/acsnano.2c08098, PMID: 36166667

[199] Huang Y Lai H Jiang J Xu X Zeng Z Ren L. pH-activatable oxidative stress amplifying dissolving microneedles for combined chemo-photodynamic therapy of melanoma. Asian J Pharm Sci. (2022) 17:679–96. doi: 10.1016/j.ajps.2022.08.003, PMID: 36382300 PMC9640714

[200] Song C Zhang X Cao Z Wei Z Zhou M Wang Y. Regulating tumor cholesterol microenvironment to enhance photoimmunotherapy in oral squamous cell carcinoma. Chem Eng J. (2023) 462:142160. doi: 10.1016/j.cej.2023.142160

[201] Zhao X Li X Zhang P Du J Wang Y. Tip-loaded fast-dissolving microneedle patches for photodynamic therapy of subcutaneous tumor. J Controlled release. (2018) 286:201–9. doi: 10.1016/j.jconrel.2018.07.038, PMID: 30056119

[202] Donnelly RF Morrow DI McCarron PA Woolfson AD Morrissey A Juzenas P. Microneedle-mediated intradermal delivery of 5-aminolevulinic acid: potential for enhanced topical photodynamic therapy. J Controlled Release. (2008) 129:154–62. doi: 10.1016/j.jconrel.2008.05.002, PMID: 18556084

[203] Diment LE Thompson MS Bergmann JH. Clinical efficacy and effectiveness of 3D printing: a systematic review. BMJ Open. (2017) 7:e016891. doi: 10.1136/bmjopen-2017-016891, PMID: 29273650 PMC5778284

[204] Yan Q Dong H Su J Han J Song B Wei Q. A review of 3D printing technology for medical applications. Engineering. (2018) 4:729–42. doi: 10.1016/j.eng.2018.07.021

[205] McGuigan AP Sefton MV. Design criteria for a modular tissue-engineered construct. Tissue Eng. (2007) 13:1079–89. doi: 10.1089/ten.2006.0245, PMID: 17439395

[206] Heisler DB Johnson KA Ma DH Ohlson MB Zhang L Tran M. A concerted mechanism involving ACAT and SREBPs by which oxysterols deplete accessible cholesterol to restrict microbial infection. Elife. (2023) 12:e83534. doi: 10.7554/eLife.83534.sa2, PMID: 36695568 PMC9925056

[207] Ranjan R Kumar D Kundu M Moi SC. A critical review on Classification of materials used in 3D printing process. Materials today: Proc. (2022) 61:43–9. doi: 10.1016/j.matpr.2022.03.308

